# Protein interaction networks in the vasculature prioritize genes and pathways underlying coronary artery disease

**DOI:** 10.1038/s42003-023-05705-1

**Published:** 2024-01-12

**Authors:** Qiuyu Martin Zhu, Yu-Han H. Hsu, Frederik H. Lassen, Bryan T. MacDonald, Stephanie Stead, Edyta Malolepsza, April Kim, Taibo Li, Taiji Mizoguchi, Monica Schenone, Gaelen Guzman, Benjamin Tanenbaum, Nadine Fornelos, Steven A. Carr, Rajat M. Gupta, Patrick T. Ellinor, Kasper Lage

**Affiliations:** 1 Cardiovascular Disease Initiative & Precision Cardiology Laboratory, Broad Institute of MIT and Harvard, Cambridge, MA USA; 2 Cardiovascular Research Center, Massachusetts General Hospital, Boston, MA USA; 3 Center for Genomic Medicine, Massachusetts General Hospital, Boston, MA USA; 4 Stanley Center for Psychiatric Research, Broad Institute of MIT and Harvard, Cambridge, MA USA; 5 Novo Nordisk Foundation Center for Genomic Mechanisms of Disease, Broad Institute of MIT and Harvard, Cambridge, MA USA; 6 Department of Surgery, Massachusetts General Hospital, Boston, MA USA; 7 Wellcome Centre for Human Genetics, Nuffield Department of Medicine, University of Oxford, Oxford, UK; 8 Genomics Platform, Broad Institute of MIT and Harvard, Cambridge, MA USA; 9 Proteomics Platform, Broad Institute of MIT and Harvard, Cambridge, MA USA; 10 Divisions of Cardiovascular Medicine and Genetics, Brigham and Women’s Hospital, Boston, MA USA; 11 Institute of Biological Psychiatry, Mental Health Centre Sct. Hans, Mental Health Services Copenhagen, Roskilde, Denmark

**Keywords:** Coronary artery disease and stable angina, Protein-protein interaction networks, Genome-wide association studies

## Abstract

Population-based association studies have identified many genetic risk loci for coronary artery disease (CAD), but it is often unclear how genes within these loci are linked to CAD. Here, we perform interaction proteomics for 11 CAD-risk genes to map their protein-protein interactions (PPIs) in human vascular cells and elucidate their roles in CAD. The resulting PPI networks contain interactions that are outside of known biology in the vasculature and are enriched for genes involved in immunity-related and arterial-wall-specific mechanisms. Several PPI networks derived from smooth muscle cells are significantly enriched for genetic variants associated with CAD and related vascular phenotypes. Furthermore, the networks identify 61 genes that are found in genetic loci associated with risk of CAD, prioritizing them as the causal candidates within these loci. These findings indicate that the PPI networks we have generated are a rich resource for guiding future research into the molecular pathogenesis of CAD.

## Abstract (web summary)

Protein-protein interaction networks of coronary artery disease (CAD) risk genes in human vascular cells are enriched for genetic variants associated with CAD and can be used to guide future research into the molecular pathogenesis of CAD.

## Introduction

CAD is the leading global cause of morbidity and mortality with high heritability^1^. While large-scale population-based association studies have identified many genetic risk loci for CAD, key challenges exist in understanding how and which genes within these loci contribute to CAD pathogenesis^2^. Despite the widely established role of lipid metabolism in CAD, most CAD-risk loci are unrelated to traditional lipid risk factors but instead point to arterial wall-specific processes^3,4^. Therefore, exploring the “non-lipid” pathways implicated by CAD genetic signals may unlock opportunities for new therapeutics.

Mass spectrometry-based interaction proteomics enables systematic mapping of PPIs in a quantitative, scalable, and cell-type-specific manner and offers great potential for establishing mechanistic connections from disease risk genes to function. Indeed, numerous studies have used PPI data to functionally interpret results from large-scale genetic studies and elucidate the etiology of complex diseases such as arrhythmia and type 2 diabetes^5–7^. However, little is known about the cell-type-specific PPIs of CAD-risk genes within the vascular tissue, and how these PPIs may represent biological pathways and networks that contribute to CAD pathogenesis.

In the current work, we sought to characterize the protein interactomes of the non-lipid CAD-risk genes in the most disease-relevant tissue, the human vasculature. We performed immunoprecipitation experiments coupled with mass spectrometry (IP-MS) for 11 CAD-risk genes in two primary human vascular cell types, endothelial cells or smooth muscle cells, and constructed cell-type-specific PPI networks using 20 high-quality IP-MS datasets. By integrating the PPI networks with other data types, we show that they contain extensive interactions that have not been reported in the literature, capture both cell-type-specific and shared biology across the two cell types, and are enriched for genetic risks of CAD-related phenotypes. Therefore, the PPI networks can be used to prioritize causal candidate genes within CAD-risk loci, provide insights into the non-lipid CAD pathogenesis, and nominate promising targets for further mechanistic and therapeutic studies.

## Results

### Study design and quality control

**Fig. 1:**
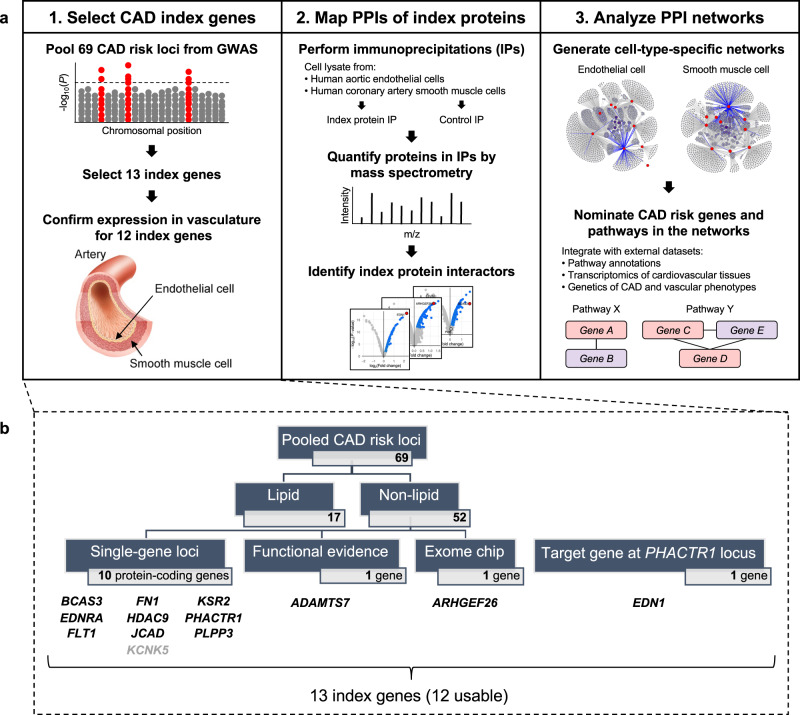
Mapping protein-protein interactions (PPIs) of coronary artery disease (CAD)-risk genes in human vascular cells. **a** Overview of the 3-stage study workflow consisting of: (1) selection of index genes in CAD-risk loci pooled from genome-wide association studies (GWAS); (2) mapping PPIs of the corresponding index proteins using immunoprecipitation coupled with mass spectrometry (IP-MS); and (3) analysis of the resulting PPI networks to uncover CAD-relevant biology. **b** Details on the selected index genes, which include ten genes from non-lipid, single-gene loci (all expressed in vascular cells except *KCNK5*), 1 gene with a confirmed causal functional role in CAD (*ADAMTS7*), 1 gene nominated by exome chip data (*ARHGEF26*), and 1 gene identified as a distal regulatory target of the *PHACTR1* locus (*EDN1*). Artery image by courtesy of Encyclopædia Britannica, Inc., copyright 2007; used with permission.

We designed a three-stage study to (1) select high-confidence CAD-risk genes (termed “index genes”) from CAD-risk loci; (2) map the PPIs of the corresponding “index proteins” in vascular cells by IP-MS experiments; and (3) integrate the resulting PPI networks with other data types to uncover CAD-relevant biology (Fig. 1a).

To select the index genes in Stage 1, we first aggregated a comprehensive list of 69 CAD-associated loci that reached genome-wide significance in the CARDIoGRAM GWAS^8^, C4D GWAS^9^, CARDIoGRAMplusC4D Metabochip^10^, CARDIoGRAMplusC4D 1000 Genomes-based GWAS^11^, or Myocardial Infarction Genetics and CARDIoGRAM Exome study^12^ (Fig. 1b and Supplementary Data 2). We removed 17 loci with known roles in regulating traditional lipid risk factors to focus on the non-lipid aspects of CAD. Among the remaining 52 non-lipid loci, we identified 12 protein-coding genes that are the likely CAD-causal candidates to be taken forward to subsequent proteomic experiments. These include ten genes from single-gene loci (Supplementary Data 2), 1 gene with a known causal role in CAD (*ADAMTS7*)^13^, and one gene with exome evidence and functional support (*ARHGEF26*)^14^. Additionally, we included a target gene that is under distal regulation of the *PHACTR1* locus, *EDN1*, which has been nominated as a CAD-causal gene with strong biological plausibility^15^. Among the 13 index genes, endogenous expression in the vasculature was confirmed for all except *KCNK5* (Supplementary Data 3), yielding 12 usable index proteins for Stage 2 of the study (Fig. 1b). An orthogonal survey of the literature on the 12 index genes showed extensive genetic and experimental evidence supporting that all these index genes are indeed implicated in the pathogenesis of CAD (Supplementary Data 4).

In Stage 2, we used each of the index proteins as bait to perform co-immunoprecipitation (co-IP) experiments in primary human aortic endothelial cells (HAEC; EC, hereafter) and human coronary artery smooth muscle cells (HCASMC; SMC, hereafter), followed by tandem mass spectrometry (MS) to identify and quantify proteins in the index protein IPs (or “bait IPs”) relative to control IPs (Fig. 1a). We used Genoppi^16^ to perform quality control (QC) and analyze data from >60 IP-MS experiments, identifying significant protein interactors of the index protein in each experiment (i.e., proteins with log_2_ fold change [FC] >0 and false discovery rate [FDR] ≤0.1 in bait vs. control IPs). We identified a subset of 20 high-quality IP-MS datasets for 11 index proteins (ADAMTS7, ARHGEF26, BCAS3, EDN1, EDNRA, FLT1, FN1, HDAC9, JCAD, PHACTR1, PLPP3), in which the replicate log_2_ FC correlation was >0.6 and the index protein itself was significant at log_2_ FC >0 and FDR ≤0.1, and restricted all subsequent analyses to these datasets (Supplementary Figs. 1–3 and Supplementary Data 5 and 6). Although some of the 20 datasets were generated under variable experimental conditions (i.e., different IP approaches, MS facilities, or cell types), an examination of their QC metrics confirmed that they were overall technically robust and comparable despite these differences (Supplementary Figs. 4–6).

### IP-MS data of JCAD yield mechanistic insights to vascular biology

The individual IP-MS datasets we generated could link the respective index proteins to undiscovered biology through newly identified interactions. As an example, we highlight the IP of endogenous JCAD performed in EC, in which the log_2_ FC correlation between IP replicates is 0.823, and JCAD itself is one of the most enriched proteins (log_2_ FC = 1.81 and FDR = 1.40e-3; Supplementary Fig. 3h and Supplementary Data 5 and 6). Out of the 35 significant interactors identified in this dataset, only one (FLNC) had been reported in PPI databases, including InWeb^17^, BioPlex^18^, iRefIndex^19^, HuRI^20^, STRING^21^, and PCNet^22^, illustrating the potential for biological discovery using our approach.

Prior studies showed that JCAD regulates Hippo signaling in endothelial cells^23^; reports from non-vascular cells further implied that the interaction between the PY motif of JCAD and the WW domain of Hippo proteins may underlie its role in Hippo signaling^24,25^. Surprisingly, in our JCAD IP-MS results, while several WW domain-containing proteins were detected, none were identified as significant interactors of JCAD (Supplementary Fig. 7), suggesting that the reported interactions between JCAD and the WW domains of Hippo proteins may not drive the specific role of JCAD in endothelial Hippo signaling. In contrast, among the 35 significant interactors of JCAD in EC, 9 (25.7%) are either centrosomal proteins or proteins with known roles in cytokinesis (Supplementary Fig. 7). Pathway analyses of the JCAD interactors also revealed significant enrichment of GO terms related to centrosomal components and cell cycle (Supplementary Data 7). These results strongly support the role of JCAD in endothelial cell proliferation, a key phenotype related to vascular injury response, including atherosclerosis. Importantly, this critical insight has been experimentally corroborated by several previous studies, which reported reduced proliferation and angiogenesis upon targeted disruption of JCAD in endothelial cells^23,26^. Together, these results support the hypothesis that the interaction between JCAD and the centrosomal proteins may connect endothelial dysfunction to CAD pathogenesis.

### Construction of de novo cell-type-specific PPI networks in human vasculature

**Fig. 2:**
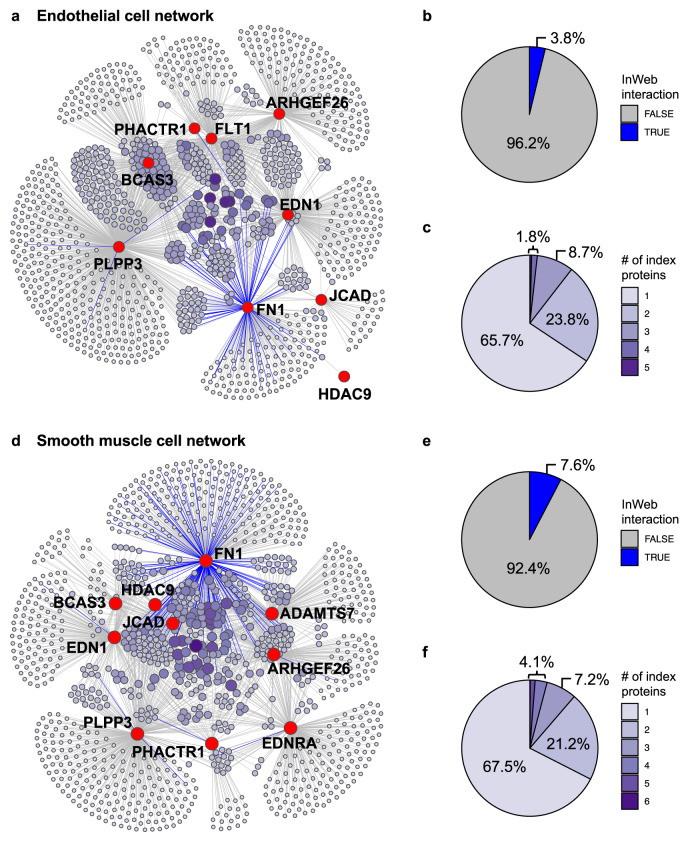
Combined PPI network in endothelial or smooth muscle cells. **a** The combined PPI network of 9 index proteins derived from IP-MS experiments in endothelial cells. Index proteins and their interactors are shown as red and purple nodes, respectively, and the edges between them indicate significant interactions in the IPs. The size and color of the interactor nodes indicate interactor frequency (i.e., the number of index proteins linked to each interactor), with larger and darker nodes representing more recurrent interactors. Color of the edges indicates whether each interaction is a known interaction in InWeb (blue) or a potentially novel interaction not found in InWeb (gray). **b** Distribution of InWeb vs. non-InWeb interactions in the network in (**a**). **c** Distribution of interactor frequency in the network in (**a**). **d**–**f** Characteristics of the combined PPI network of 10 index proteins derived from IP-MS experiments in smooth muscle cells. The same legends for (**a**–**c**) apply here.

In Stage 3 of our study, we assembled the 20 high-quality IP-MS datasets into cell-type-specific PPI networks and intersected them with other data types to extract biological insights (Fig. 1a). First, we generated the combined PPI networks for EC and SMC using all IP-MS data derived from the respective cell type (Fig. 2a, d and Supplementary Data 8). The EC network contains 9 index proteins and 1190 significant interactors, while the SMC network contains 10 index proteins and 1122 interactors. Over 90% of the interactions in our data have not been reported in the literature according to InWeb (Fig. 2b, e) and 5 other PPI databases (Supplementary Fig. 8 and Supplementary Data 8) and thus represent potentially novel biology. Furthermore, there is substantial convergence among the interactomes of individual index proteins in both cell types, with >30% of the interactors being linked to multiple index proteins (Fig. 2c, f) and many index proteins sharing a significant number of common interactors (Fig. 2a, d and Supplementary Data 9). In fact, we observed several interactions between the index proteins themselves: FN1 was identified as an interactor of EDN1 in SMC and of PLPP3 in both cell types. Such convergent patterns suggest that some of the index proteins may participate in common vascular pathways or recurring processes that are CAD-relevant yet need to be defined functionally.

Importantly, the interactions in our de novo PPI networks are experimentally reproducible. For instance, we have validated the interactions of several recurrent interactors (i.e., interactors linked to multiple index proteins) identified in both EC and SMC, including PDIA6, RPL7A, and HSPA9 (Supplementary Data 8), in individual IPs followed by western blotting (IP-WB; Supplementary Fig. 9**)**. In parallel, we also performed reciprocal IPs in SMC using several newly discovered protein interactors of ADAMTS7 and JCAD as baits, and successfully detected the presence of ADAMTS7 or JCAD in these reciprocal IPs by western blot (Supplementary Fig. 10). Overall, our validation results are comparable with previous reports that demonstrate up to ~90% validation rate for PPIs identified by IP-MS^16,27,28^, and indicate that our vascular PPI networks contain high-confidence interactions.

### Shared and cell-type-specific PPIs in endothelial cells vs. smooth muscle cells

**Fig. 3:**
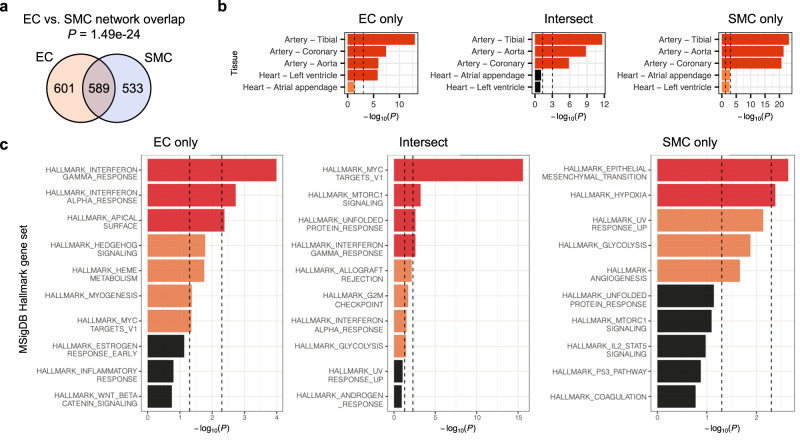
Comparison of PPIs in the endothelial cell (EC) vs. smooth muscle cell (SMC) networks. **a** Overlap between interactors in the EC and SMC PPI networks. **b** Cardiovascular tissue enrichment calculated using GTEx tissue-specific genes based on RNA-seq data. Interactors found exclusively in the EC (EC only) or SMC (SMC only) network and interactors found in both networks (Intersect) were analyzed separately and compared against the rest of the genome. **c** Gene set enrichment calculated using MSigDB Hallmark gene sets. EC only, SMC only, and Intersect interactors were compared against the non-interactors detected by IP-MS; only the top ten gene sets are shown for each analysis. All *P* values were calculated using one-tailed hypergeometric tests. For (**b**, **c**), nominally (*P*<0.05) or Bonferroni-significant (*P* <0.05/number of tissues or gene sets) results are shown in orange or red, respectively; gene counts used for analysis are shown in Supplementary Data 10 and 11.

We compared the PPIs observed in endothelial cells vs. smooth muscle cells to identify common or cell-type-specific CAD-relevant biology. Globally, about half of all protein interactors from the EC and SMC networks are shared by the two cell types (49.5% in EC and 52.5% in SMC; Fig. 3a). While this overlap is statistically significant (*P* = 1.49e-24), it also indicates that ~50% of the interactors are specific to one cell type but not the other. To explore this in more detail, we stratified the overlap analysis by each of the 8 index proteins that have IP-MS data in both cell types (ARHGEF26, BCAS3, EDN1, FN1, HDAC9, JCAD, PHACTR1, PLPP3), and found significant overlap between the EC and SMC interactors of FN1 (*P* = 1.59e-8), PHACTR1 (*P* = 0.0317), and PLPP3 (*P* = 3.52e-12), but not for the other five index proteins (Supplementary Fig. 11 and Supplementary Data 9**)**. Our observations are in line with publications demonstrating distinct roles in EC vs. SMC for ARHGEF26^29,30^, BCAS3^31^, EDN1^32^, JCAD^33^, and HDAC9^34–36^, but partially shared functions in EC and SMC for FN1 (to modulate extracellular matrix^37^) and PLPP3 (to attenuate inflammation and permeability following vascular injury^38,39^). Together, these results highlight both the functional commonality represented by the overlapping interactors, as well as the divergent roles of cell-type-specific interactors in the two cell types.

### Vascular PPI networks are enriched for tissue types and pathways related to CAD

One way to explore the causal mechanisms implicated by the PPI networks is to examine whether the networks are enriched for genes specifically expressed in disease-relevant tissues or cell types. Therefore, we assessed the overlap enrichment between interactor genes in the networks and tissue-specific genes derived from RNA sequencing data of GTEx tissues^40^. Both the EC and SMC networks are significantly enriched (*P* < 0.05/53, adjusting for 53 tissues) for genes specific to cardiovascular tissues, and as expected, tissues containing rich SMC (e.g., digestive organs and uterus; Supplementary Fig. 12a and Supplementary Data 10). Notably, genes specific to adipose tissue are also significantly enriched, which highlights the indispensable role of adipose tissue in vascular homeostasis that are tightly coupled to EC and SMC^41,42^. To further compare the EC and SMC networks, we analyzed sub-networks consisting of interactors found in only one cell type (“EC only” or “SMC only”) or interactors shared by both cell types (“Intersect”; Supplementary Fig. 12a and Supplementary Data 10). Among the cardiovascular tissues, all three sub-networks show significant enrichment for genes specific to aortic, coronary, and tibial artery tissues, while the “EC only” network is additionally enriched for genes specific to left ventricle tissue (Fig. 3b). Since mRNA and protein abundance show variable correlation across tissues^43^, we also repeated the analysis using tissue-specific genes defined from proteomic data of GTEx tissues^44^. The results derived from these data have weaker significance overall but show similar enrichment patterns in the cardiovascular tissues (Supplementary Figs. 13a, 14 and Supplementary Data 10). These findings reaffirm that our de novo PPI networks point to vascular-specific genes and further support the use of both cell types to understand the genetic basis of CAD.

We next assessed whether our PPI networks are enriched for biological pathways represented by the MSigDB^45,46^ Hallmark and Reactome gene sets and Gene Ontology^47,48^ (GO) terms. In these pathway analyses, instead of comparing the interactor genes in our networks to the rest of the genome, we compared them to other genes that were detected in our IP-MS experiments (i.e., the “non-interactors” in Supplementary Data 8). We reasoned that since both the interactors and non-interactors show elevated protein expression in human heart cell types^49^ (Supplementary Fig. 15) and are enriched for tissue-specific genes in cardiovascular tissues (Supplementary Figs. 12 and 13 and Supplementary Data 10), comparing the interactors against the non-interactors would allow us to assess the conditional enrichment of the networks in a way that accounts for the cellular context of our data. In the MSigDB Hallmark analysis, the “EC only” and “Intersect” networks show significant (*P* <0.05/50, adjusting for 50 gene sets) or nominal (*P* <0.05) enrichment for similar gene sets, including “MYC targets” and immunity-related pathways (“interferon gamma response”, “interferon alpha response”, and “allograft rejection”; Fig. 3c and Supplementary Data 11). In contrast, the “SMC only” network is most enriched for processes broadly related to the arterial wall, including “epithelial mesenchymal transition”, “hypoxia”, and “angiogenesis”. In the Reactome and GO analyses, we also observed some divergent patterns between these networks (Supplementary Fig. 16 and Supplementary Data 11 and 12). Overall, the tissue and pathway enrichment results show that the EC and SMC PPI networks capture both shared and cell-type-specific biology related to CAD.

### Linking vascular PPI networks to genetic risks of CAD and related phenotypes

**Fig. 4:**
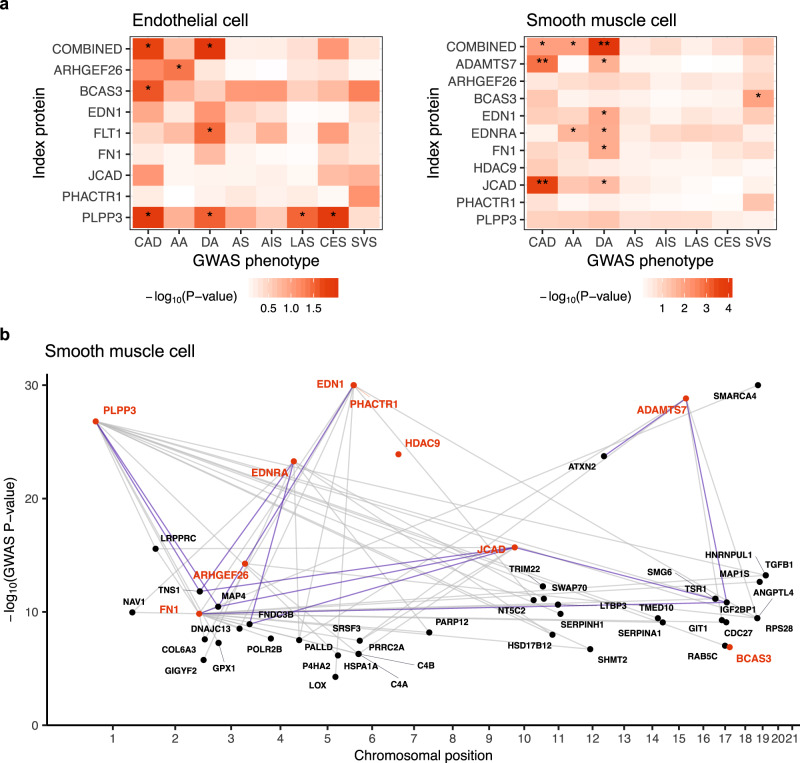
Genetic risk enrichment in the PPI networks. **a** Common variant enrichment of the PPI networks calculated using MAGMA and GWAS summary statistics of CAD, aorta size, and stroke. Index protein interactors identified in EC or SMC were compared against the rest of the protein-coding genome. Nominal (*P*<0.05) or Bonferroni (*P*<0.05/29) significance is indicated by single or double asterisks, respectively; gene counts used for analysis are shown in Supplementary Data 13. AA ascending aorta, DA descending aorta, AS any stroke, AIS any ischemic stroke, LAS large-artery atherosclerotic stroke, CES cardioembolic stroke, SVS small-vessel stroke. **b** Social Manhattan plot of genes encoding the index proteins (red) and their SMC interactors (black) in genome-wide significant CAD GWAS loci. Links between genes indicate observed protein-protein interactions; interactions validated by western blots are highlighted in blue.

To assess whether the PPI networks are associated with genetic risk factors of CAD, we used MAGMA^50^ to evaluate the genetic risk enrichment within the networks relative to other protein-coding genes (“global” analysis) or to the non-interactors identified by IP-MS (“conditional” analysis). Using CAD GWAS summary statistics from a meta-analysis of the UK Biobank and CARDIoGRAMplusC4D^51^, we found the ADAMTS7 (*P* < 1.37e-3) and JCAD (*P* < 3.11e-4) networks in SMC to be significantly enriched (*P* < 0.05/29, adjusting for 29 networks) for CAD risk in the global analysis (Fig. 4a, Supplementary Fig. 17a, and Supplementary Data 13). In the more conservative conditional analysis, the ADAMTS7 network remained nominally significant, suggesting that the observed enrichment signal is robust and that genes in this network may confer risk above what one would expect for genes generally expressed in SMC (Supplementary Fig. 17b). Indeed, ADAMTS7 mediates vascular SMC migration and neointimal formation in animal carotid artery injury models, and the CAD-risk coding variant rs3825807 within the *ADAMTS7* locus affects patient-derived vascular SMC migration^52–54^. For JCAD, its interactors in SMC have no overlap with those identified in EC; thus the enrichment of CAD-risk GWAS signal among JCAD interactors appears to be specific to SMC (Supplementary Fig. 17a). Corroborating with this MAGMA result are the observations that JCAD is expressed in vascular SMC^55^, and that depletion of JCAD inhibited vascular maturation by depleting SMC in neovessels^26^. Although a role of JCAD in endothelial cells has been connected to atherosclerosis^33,55^, its role in vascular SMC has been less well understood. Our data support the role of JCAD in vascular SMC that may be critical to CAD.

We also performed analogous MAGMA analyses using GWAS summary statistics of other vascular phenotypes, including aortic size^56^ (ascending thoracic aortic diameter (AA), descending thoracic aortic diameter (DA)) and stroke subtypes^57^ (any stroke (AS), any ischemic stroke (AIS), large-artery atherosclerotic stroke (LAS), cardioembolic stroke (CES), small-vessel stroke (SVS)). We found the combined PPI network in SMC to be significantly enriched (*P* = 8.77e-5) for genetic variants associated with DA in the global analysis (Fig. 4a, Supplementary Fig. 17a, and Supplementary Data 13). The pathogenic basis of aortic aneurysm differs between ascending and descending aortas. Atherosclerosis is the predominant driving etiology leading to aneurysms of the descending aorta, but rarely causes ascending aortic aneurysms^58,59^. Therefore, the significant enrichment for genetic variants associated with DA in the SMC network highlights the cell type and proteins that may contribute to both CAD and descending thoracic aneurysms. In fact, among the index proteins whose networks show nominal enrichment for DA, ADAMTS7^60^ and EDN1^61,62^ have been linked to aortic aneurysms in previous studies. Taken together, we observed enrichment of CAD-risk GWAS variants among the ADAMTS7 and JCAD PPI networks derived from SMC, and an association between the combined SMC PPI network and descending aortic size. As better-powered GWAS datasets become available, the suggestively significant enrichment for other networks and phenotypes reported here could be validated in the future.

### Using vascular PPI networks to prioritize candidate CAD-risk genes

After establishing that some of our PPI networks are enriched for genetic risks of CAD-related phenotypes, we used the networks to prioritize additional CAD-risk genes from GWAS data. Given that the evidence for the causal gene(s) within a GWAS locus are often absent, ambiguous, or conflicting, physical interactions with known or high-confidence risk genes may serve as an important functional indicator of the potentially causal gene(s) for a given locus. When we intersected our PPI networks with genes found in genome-wide significant GWAS loci for CAD^51^, we found that the index proteins in the combined EC network interact with 43 proteins encoded by genes in the CAD-risk loci (termed “locus proteins”), while the index proteins in the SMC network are linked to 41 locus proteins (Fig. 4b, Supplementary Fig. 18a, and Supplementary Data 14). Together, our PPI data prioritize 61 unique genes within CAD-risk loci across the two cell types. We confirmed the reproducibility of a subset of the PPIs between index and locus proteins by independent IP-WB or reciprocal IP-WB (Supplementary Figs. 10 and 19). For instance, we were able to validate several interactions involving IGF2BP1, MAP4, and TNS1, which are all located within CAD-risk loci containing multiple candidate genes and are also found to be recurring interactors in our networks.

When selecting the index proteins in this study, we included two index proteins from the multi-gene chromosome *6p24* locus, which contains *EDN1* and *PHACTR1* in a 1-Mb region around the sentinel variant rs9349379^15^. There is uncertainty regarding which of these genes is causal for the multiple vascular diseases associated with this locus. Therefore, we compared the overlaps between genes in GWAS loci and EDN1 or PHACTR1 interactomes in vascular cells to see if they could help prioritize one of the genes over the other. We observed that EDN1 interacts with many more locus proteins compared to PHACTR1 in both EC and SMC (Supplementary Fig. 18b and Supplementary Data 14). Across both cell types, EDN1 is linked to 11 locus proteins and 1 other index protein (FN1), while PHACTR1 is only associated with 2 locus proteins that are also EDN1 interactors. There is substantial evidence supporting the critical roles of several locus proteins that interact with EDN1 in the vasculature, including MAP4^63^, SRSF3^64^, LOX^65–67^, and TOP1^68^. Furthermore, consistent with the known role of EDN1 in mediating proliferation and vasoconstriction in smooth muscle cells, its interactors in SMC include many extracellular matrix proteins (i.e., COL6A3, FN1, and LOX). Finally, we also mapped the PPIs of the major receptor for EDN1, EDNRA, in SMC: we found EDNRA to interact with 8 locus proteins, including PALLD, which is also an interactor of EDN1 in SMC (Fig. 4b and Supplementary Data 14). In agreement with these observations, both the EDN1 and EDNRA PPI networks in SMC show nominally significant enrichment for aortic size in our genetic analysis, while the PHACTR1 network shows no enrichment (Fig. 4a). Together, these findings support the hypothesis that EDN1, rather than PHACTR1, is a more likely driver of the GWAS signal for CAD risk observed in the *6p24* locus.

The recent development of large-scale CRISPR perturbation screens have allowed experimental validation of plausible causal genes in a high-throughput, unbiased manner. To further assess the functional relevance of the index and locus proteins prioritized by our PPI data in the context CAD, we examined data from a recent study that performed pooled CRISPR screens targeting CAD GWAS loci in immortalized human aortic endothelial cells (teloHAEC)^69^. The study used CRISPR knockout, inhibition, and activation to target 1998 potential causal variants in 83 CAD loci and identified 26 loci significantly associated with endothelial phenotypes related to CAD. Five of these significant loci mapped to genes (*FGD5, LOX, MAT2A, NT5C2, SMG6*) that were also prioritized by our PPI data (Fig. 4b, Supplementary Fig. 18a, and Supplementary Data 15). Specifically, perturbing the variants in these loci affected the levels of adhesion proteins (E-Selectin, ICAM1, VCAM1) and/or signaling molecules (nitric oxide, reactive oxygen species, calcium) in endothelial cells, which have all been directly implicated in the pathology of CAD. In line with the positive CRISPR screen hits and the nomination by our PPI network, there is the abundance of genetic and experimental evidence directly implicating FGD5^70^, LOX^71,72^, MAT2A^73^, NT5C2^74–76^, and SMG6^8,77–79^ in CAD pathogenesis. These results provide concrete examples of how combining our PPI-nominated candidate proteins with phenotypic perturbation screens can help accelerate rapid functional validation of candidate CAD-causal genes in disease-relevant cell types.

## Discussion

The success of large-scale, population-based association studies in mapping susceptibility loci for CAD has been eclipsed by the herculean efforts to pinpoint the causal genes within these loci and to understand their biological and clinical relevance. To help fill in the gaps in the “variant-to-function” relationships, we performed interaction proteomics to map the PPIs of 11 non-lipid CAD-risk genes in two disease-relevant vascular cell types, endothelial and smooth muscle cells. The resulting PPI networks capture both cell-type-specific and shared biology between the two cell types and overlap with genetic signals of CAD and related vascular phenotypes. These results demonstrate the capacity of using PPIs to dissect the genetic basis of CAD and indicate that our PPI data can serve as a rich resource for accelerating the translation of GWAS signals into biological insights.

A particular strength of our work lies in our vascular-specific approach, as mapping pathogenic processes in defined primary cells could offer new insights into the molecular basis of disease^80^. For CAD, understanding the tissue-specific pathology holds translation value for highly specific intervention strategies to target organs, as demonstrated by recent development of gene editing in the liver to lower cholesterol^81^ or gene silencing in the vasculature to suppress endothelial genes^82^. Furthermore, given that most CAD-risk loci are unrelated to lipid risk factors, understanding the non-lipid pathways of CAD is imperative for developing novel, efficacious therapeutics. Therefore, mechanistic insights inspired by this work will be an informative first step to begin functionally annotating the non-lipid CAD susceptibility loci that are poorly understood.

Our findings need to be interpreted with its limitations. First, our PPI networks were derived from only 11 index proteins that can be confidently linked to CAD in two vascular cell types, and therefore represent only a small fraction of CAD biology. As the fuller spectrum of genotypes and CAD phenotypes are becoming available through population-based biobanks such as the UK Biobank, the framework described here can be applied to generate broader PPI networks with substantially higher scale and resolution in the future. In addition, the studied index proteins may have CAD-relevant roles outside of the vasculature (e.g., ADAMTS7 is a secreted enzyme) that are not accounted for by the PPI networks derived from vascular cell lysates.

Second, the IP-MS approach for identifying PPIs has various caveats that may influence the reproducibility of the data. For instance, the quality of IP antibodies, overexpression of FLAG-tagged proteins, and incomplete coverage of proteins during MS analysis^83,84^ could all contribute to variability in the IP-MS experiments. We partially accounted for this issue by using two bait vs. control IP replicates in each IP-MS experiment to define statistically significant protein interactions. We independently replicated a subset of the interactions by western blotting (Supplementary Figs. 9, 10, and 19) and observed significant overlaps between several IPs for the same index protein (Supplementary Fig. 11), both of which support a degree of robustness in our data. However, other IPs for the same index protein have limited agreement, either due to experimental variability or due to true biological differences between cell types. Moreover, it is important to note that even when an interaction is reproducible biochemically, additional experiments beyond IP-MS will be needed to investigate if it plays a functional role in biological processes. Therefore, the putative CAD-relevant PPIs, genes, and pathways nominated by our data require further replication and functional validation before causal links to vascular biology can be established.

Third, we recognize that methods such as CRISPR perturbation screens will be crucial for the systematic functional validation of our results. Reassuringly, recent CRISPR knockout, inhibition, and/or activation experiments in immortalized teloHAEC^69^ already linked several of our prioritized CAD-risk genes to endothelial cell phenotypes, providing orthogonal support that these PPI network genes may be involved in CAD-relevant biology (Supplementary Data 15). However, this kind of in vitro perturbation approaches also has fundamental caveats that need to be considered when designing a systematic validation experiment, including the inherently variable efficiency of gene inhibition/activation, the poorly characterized off-target effect, and most importantly, the lack of correlation between protein expression level and the mRNA level of particular genes. Specific to our work, there are also considerable transcriptomic differences between the primary HAEC used in our experiments and the immortalized teloHAEC used in the CRISPR screens, and CRISPR gene editing in primary human vascular SMC has not been amenable. Thus, while CRISPR technology represents a promising avenue for functional validation of our PPI networks in human cell models, it is beyond the scope of the current study and warrants a separate effort to properly leverage its strengths in the future.

In conclusion, our work showcases how cell-type-specific interaction proteomics is a powerful approach for characterizing CAD-risk genes in an unbiased, scalable fashion. Genes and pathways prioritized by our vascular-specific PPI networks can provide initial clues on how particular genetic risk factors may lead to CAD and other vascular pathology, thereby nominating potential therapeutic targets for functional validation studies. Lastly, going beyond CAD, functional PPI networks can serve as a general framework for systematic prioritization of candidate genes in GWAS loci of complex diseases.

## Methods

### Cell culture

To resemble the tissue basis for CAD, primary human coronary artery endothelial cells (HCAEC) and smooth muscle cells (HCASMC) were the preferred cell types. However, our preliminary study showed that HCAEC lacks sufficient proliferative capacity to support scalable yield of proteomic samples, particularly with regard to epitope-tagged index protein production by expression vector transfection. As an alternative, we identified a more proliferative EC type with a transcriptional profile that resembles HCAEC, human aortic endothelial cell (HAEC)^85^. HAEC and HCASMC were then used to carry out all proteomic experiments.

HAEC and HCASMC from multiple healthy donors were pooled and maintained in VascuLife EnGS and SMC media, respectively (cell and medium purchased from Lifeline Cell Technology), and used at passage <8 for all experiments. HEK293 cell was from ATCC and maintained in high-glucose Dulbecco’s Modified Eagle Medium (DMEM) with GlutaMAX supplement and 10% fetal bovine serum (FBS; Thermo Fisher Scientific). All cell culture was maintained free of antibiotics in a humidified incubator at 37 °C with 5% CO_2_.

### Selection of index proteins

We aggregated 69 genetic loci that have been associated with CAD from CARDIoGRAM GWAS^8^, C4D GWAS^9^, CARDIoGRAMplusC4D Metabochip^10^, CARDIoGRAMplusC4D 1000 Genomes-based GWAS^11^, and Myocardial Infarction Genetics and CARDIoGRAM Exome study^12^ (Supplementary Data 2). We noted 17 loci were located near genes with known roles in regulating traditional lipid risk factors for CAD: LDL, triglyceride-rich lipoproteins, or lipoprotein(a), and may therefore be contributing to CAD-risk via the well-studied lipid metabolism^1^. These loci were removed, leaving 52 “non-lipid” CAD-risk loci that are likely to represent vascular-specific pathways of CAD pathogenesis. Next, we defined linkage disequilibrium (LD) boundaries for the leading CAD-risk SNP in each of the remaining loci, which span SNPs with *r*^2^ >0.6 ± 50 kb on either end. We then searched for protein-coding genes within each LD locus to identify a subset of 13 loci containing only a single protein-coding gene (i.e., single-gene loci, or SGL). The 13 SGL contain 10 unique genes (*BCAS3*, *EDNRA*, *FLT1*, *FN1*, *HDAC9*, *JCAD*, *KCNK5*, *KSR2*, *PHACTR1*, *PLPP3*) that are possible CAD-causal genes in these loci, and thus their encoded proteins were used as the “index proteins” in our study. We also included three additional index proteins encoded by genes that have been previously implicated in CAD, including 1 gene with a known causal role in CAD (*ADAMTS7*)^13^, 1 gene with exome evidence and functional support (*ARHGEF26*)^14^, and a distal regulatory target of the *PHACTR1* locus, *EDN1*, which has been nominated as a CAD-causal gene with strong biological plausibility^15^.

We surveyed RNA-seq data generated by the ENCODE project^86^ to confirm the endogenous expression of the selected index proteins in HAEC (GEO accession: GSE78613) and HCASMC (GEO accession: GSE78534; Supplementary Data 3). We found that KCNK5 was the only index protein with no detectable RNA expression, and thus excluded it from further experiments. Furthermore, we compared the expression levels of different transcripts to identify the dominant transcript variant for each index gene, and by inference, the dominant protein isoform for each index protein. These dominant transcript variants serve as the template sequences for constructing overexpression vectors, as described below.

### Construction of mammalian expression vectors for index proteins

The cDNA containing the open-reading frame (ORF) of the endothelial *ARHGEF26* transcript (*NM_015595*) was obtained from the Mammalian Gene Collection and cloned with a 3×FLAG tag and a GGGS linker sequence into a pcDNA3.4 mammalian expression vector (Thermo Fisher Scientific). The ORF sequences carrying a 3×FLAG tag for ADAMTS7, BCAS3, EDN1, FLT1, HDAC9, PHACTR1, and PLPP3 were constructed by GeneArt Gene Synthesis (Thermo Fisher Scientific) using customized DNA constructs and cloned onto the pcDNA3.4 vector. All vector sequences have been validated by Sanger sequencing, and protein expression at the expected molecular weight was confirmed by Western blot using HEK293 cell lysate overexpressing the respective vectors. The remaining index proteins have commercially available IP-competent antibodies, and therefore do not require mammalian expression vectors.

### Overexpression of index proteins by consecutive transfection

For optimal expression of FLAG-tagged index proteins in primary cells, we performed two rounds of consecutive transfection in HAEC and HCASMC, respectively.

Transfection in HAEC was performed with 5 μg plasmid DNA per 1 × 10^6^ cells in 100 μL P5 Primary Cell Solution using an Amaxa 4D-Nucleofector (Lonza). A pcDNA3.4 vector without insert was used as empty control for the same number of cells as “mock” transfection. In total, 8–10 × 10^6^ cells at 70–80% confluence were nucleofected with the index protein vector or empty vector (mock transfection), respectively. Nucleofected HAEC was immediately plated in prewarmed Opti-MEM I reduced serum media (Thermo Fisher Scientific) for 2–3 h, followed by replacement with complete EnGS medium after cell attachment. Three days after the first round of nucleofection, cells were digested by Trypsin-EDTA (0.5%), washed in PBS, and underwent a second round of nucleofection (5 μg plasmid DNA per 1 × 10^6^ cells) and plating. HAEC was harvested 3–4 days after the second round of nucleofection.

Transfection in HCASMC was performed with Lipofectamine LTX with PLUS Reagent (Invitrogen) following the manufacturer’s instruction. Briefly, cells were plated on five 15-cm dishes 1–2 days before transfection at 70% confluency. Prior to transfection, cells were carefully rinsed with prewarmed Opti-MEM I media to reduce cell-derived polyanions that inhibit transfection, and gently replaced with 14 mL Opti-MEM I media. For each 15-cm dish, 20 µg plasmid DNA (for index protein or empty vector) was combined with 60 µL Lipofectamine LTX and 60 µL PLUS Reagent in 3.6 mL Opti-MEM I media, incubated for 5 min at room temperature, and added dropwise to each dish. HCASMC was incubated with the transfection mixture at 37 °C for 4 h, which was gently replaced by fresh, prewarmed Opti-MEM I media and incubated for another 2–3 h to terminate the transfection reaction and minimize DNA toxicity. Cells were then replaced with complete SMC medium. A second round of transfection was performed in 2–3 days with identical protocols. Complete SMC medium was replaced every 2–3 days. HCASMC was harvested 3–4 days after the second round of nucleofection.

### Co-immunoprecipitation using index proteins as baits

Co-immunoprecipitation (Co-IP) was carried out by either (1) using commercial antibodies to the endogenous index proteins, if such antibodies were proven IP-competent and target-specific by a pilot IP followed by probing the immunoprecipitant with a different antibody, or (2) pulling down of overexpressed, FLAG-tagged index proteins with an antibody against the FLAG tag, if IP-competent antibodies to endogenous proteins were unavailable. The control IP was performed as either a pull-down using normal isotype IgG (control for endogenous index proteins) or a pull-down of cell lysate receiving empty-vector transfections (“mock” transfection; control for FLAG-tagged index proteins).

Cells were lysed in ice-cold Pierce IP Lysis Buffer (Thermo Fisher Scientific) supplemented with fresh protease inhibitors (Pierce Mini Tablet, EDTA free), passed through a 25G syringe, and spun for 15 min at 21,000 × *g* at 4 °C. The supernatant was collected and normalized for protein concentration using a bicinchoninic acid (BCA) assay (Thermo Fisher Scientific). For pull-down of FLAG-tagged index proteins (i.e., baits), normalized cell lysate from bait- or mock transfection was incubated with washed anti-FLAG M2 magnetic beads (Sigma-Aldrich, M8823) or anti-FLAG M2 Affinity Agarose Gel (Sigma-Aldrich, A2220) at 1mg lysate per 25 μL beads ratio overnight at 4 °C with mixing. For pull-down of endogenous baits, cell lysate was pre-cleared by incubation with normal mouse IgG conjugated to agarose (Santa Cruz Biotechnology, sc-2343) or normal rabbit IgG (R&D Systems, AB-105-C) conjugated to Protein A/G Magnetic Beads (Pierce, 88802) at 1mg lysate per 10 μg IgG ratio for 1 h at 4 °C. The pre-cleared supernatant was then split into two equal halves that were combined with primary antibody or isotype IgG and beads (1mg lysate per 10 μg antibody/IgG) and incubated at 4 °C overnight with mixing. The sources of antibodies and normal IgG for Co-IP are listed in Supplementary Data 6.

After overnight incubation, each bait or control IP mixture was carefully split into three identical replicates using wide bore pipette tips. The supernatant was discarded, and the beads were washed once with ice-cold IP buffer, and three times with 100 mM triethylammonium bicarbonate (TEAB) buffer. Two of the three replicates were stored in 100 μL 100 mM TEAB buffer, pH 8.5, and snap-frozen until processed for mass spectrometry. The remaining one replicate was saved for quality control by eluting in 2× Laemmli Sample Buffer and boiling followed by Western blot analyses.

### Western blot

Reduced protein samples were resolved by sodium dodecyl sulfate-polyacrylamide gel electrophoresis (SDS-PAGE) on 4–20% or 8–16% Mini-PROTEAN TGX precast gels (Bio-Rad Laboratories), transferred to a nitrocellulose membrane, and blocked with 5% nonfat milk in Tris-buffered saline supplemented with 0.05% Tween-20 (TBST) at room temperature for 1 hour. The membrane was then incubated with primary antibodies in 1% nonfat milk in TBST overnight at 4 °C. To avoid interference from denatured heavy or light chains of IP antibodies eluted from pull-down samples, two approaches were employed: (1) blots for FLAG-tagged baits were detected by incubation directly with HRP-conjugated anti-FLAG primary antibody (Sigma-Aldrich, A8592) without using secondary antibodies; or (2) blots for endogenous baits were incubated with Clean-Blot IP reagent (Thermo Fisher Scientific 21230) in 1% nonfat milk in TBST for 1 h at room temperature, which specifically binds to whole IgG but not IgG fragments, except for blots where the signals from Clean-Blot IP reagent were undetectable or too weak, in which case conventional HRP-conjugated anti-rabbit (R&D Systems, HAF-008) or anti-mouse (R&D Systems, HAF-007) secondary antibodies were used. After extensive washing, the membranes were developed in an enhanced chemiluminescence substrate (EMD Millipore) and imaged on Amersham Imager 600 (GE Healthcare).

The non-FLAG primary antibodies used in Western blot were as follows: ADAMTS7 (Abcam, Ab28557), ATXN2 (Novus Biologicals, NBP1-90063), EDNRA (Abcam, ab242440), FLT1 (Thermo Fisher Scientific, PA5-16493), FN1 (Sigma-Aldrich, AB1945), FNDC3B (Novus Biologicals, NBP1-90495), HSPA9 (Cell Signaling Technology, 3593T), IGF2BP1 (Cell Signaling Technology, 8482), JCAD (Sigma-Aldrich, HPA017956), MAP4 (Proteintech, 11229-1-AP), PDIA6 (Sigma-Aldrich, HPA034652), RPL7A (Cell Signaling Technology, 2415), and TNS1 (Novus Biologicals, NBP1-84130). Reciprocal IPs of selected interactors (Supplementary Fig. 10) prior to western blot analysis were performed using the same antibodies as indicated above.

### Mass spectrometry and protein quantification (Whitehead)

#### Sample preparation

Starting with IP samples on beads supplied in 100 μL of 100 mM TEAB buffer, reduction and alkylation of disulfide bonds were carried out by addition of 2 μL of 50 mM Tris(2-carboxyethyl) phosphine (TCEP) in 100 mM TEAB (Sciex 4326685) for 60 min at 60 °C, followed by addition of 1 μL of 2% S-methyl methanethiosulphonate in isopropanol (Sciex 4352159) for 10 min at room temperature. Proteins in this solution were then digested by the addition of 250 ng of TPCK-treated trypsin in 50 mM TEAB (Sciex 4352157) and overnight incubation at 37 °C with gentle shaking. iTRAQ4-plex (Sciex) or TMT 6-plex (Thermo Fisher Scientific) reagents were resuspended in 50 µL isopropanol and added to each sample followed by vortex and spin; the specific iTRAQ or TMT labels used for each pair of bait or control IP replicates are indicated in Supplementary Data 6. The samples were combined and incubated at room temperature for 2 h, and then washed, extracted, and concentrated by solid phase extraction using Waters Sep-Pak Plus C18 cartridges. Organic solvent was removed, and the volumes were reduced to 80 μL via speed vacuum.

#### Chromatographic separations

The labeled tryptic peptides were subjected to basic (high pH) reversed-phase high-performance liquid chromatography (HPLC) with fraction collection using Shimadzu LC-20AD pumps and a FRC-10A fraction collector. Samples were loaded on a 10 cm × 2.1 mm column packed with 2.6 μm Aeris PEPTIDE XB-C18 media (Phenomenex). The initial gradient condition was isocratic 1% buffer A (20 mM ammonium formate in water, pH = 10) at 150 µL min^−1^, with increasing buffer B (acetonitrile) concentrations to 16.7% B at 20.5 min, 30% B at 31 min, and 45% B at 36 min. The column was washed with high percent B and re-equilibrated between analytical runs for a total cycle time of ~55 min. Sixteen 450 µL fractions (fx) were collected, combined into eight samples (fx1+2, fx3+9, fx4+10, fx5+11, fx6+12, fx7+13, fx8+14, fx15+16), then reduced to 20 µL via speed vacuum. The combined samples were subjected to reversed-phase HPLC using Thermo EASY-nLC 1200 pumps and autosampler, followed by mass spectrometry using a Thermo Q Exactive HF-X Hybrid Quadrupole-Orbitrap mass spectrometer and a nanoflow configuration. Samples were loaded on a 6 cm × 100 μm column packed with 10 μm ODS-A C18 material (YMC), washed with 4 μL total volume to trap and wash peptides, then eluted onto the analytical column packed with 1.7 μm Aeris C18 material (Phenomenex) in a fritted 14 cm × 75 μm fused silica tubing pulled to a 5-μm tip. The initial gradient condition was 1% buffer A (1% formic acid in water) at 300 nL min^−1^, with increasing buffer B (1% formic acid in acetonitrile) concentrations to 6% B at 1 min, 21% B at 42.5 min, 36% B at 63.15 min, and 50% B at 73 min. The column was washed with high percent B and re-equilibrated between analytical runs for a total cycle time of ~97 min.

#### Mass spectrometry

The mass spectrometer was operated in a data-dependent acquisition mode where the 20 most abundant peptides detected in the Orbitrap using full scan mode with a resolution of 60,000 were subjected to daughter ion fragmentation using a resolution of 15,000. A running list of parent ions was tabulated to an exclusion list to increase the number of peptides analyzed throughout the chromatographic run.

#### Protein quantification

Mass spectra were analyzed using PEAKS Studio X+ (Bioinformatics Solutions). For peptide and protein identification, mass spectra were searched against the *Homo sapiens* UniProtKB/TrEMBL database (release 2019_01) containing isoforms and a set of common laboratory contaminants. Positive identification was used and quantitation was based on the top three total ion current (TIC) method, with a maximum FDR of 1% at the spectrum level. Tolerance on the precursor was 10 ppm, on the fragments 0.01 Da, with carboxymethylation (C) as fixed modification and oxidation (M), deamidation (NQ), phosphorylation (STY), and acetylation (N-Ter) as variable modifications. Relative ratios of the iTRAQ or TMT reporter ions were used for protein-level quantitation across bait and control IP replicates.

### Mass spectrometry and protein quantification (Broad)

#### Sample preparation

Proteins were digested on beads using 90 µl of digestion buffer (2 M urea/50 mM Tris buffer with 1 mM DTT and 5 µg/mL Trypsin) for 1 h, shaking at 1000 rpm. The suspension was then transferred to a new tube, and the beads were washed twice with 60 µL of wash buffer (2 M urea/50 mM Tris buffer). The wash buffer was added to the suspension with digestion. The digestion and wash process was repeated a second time pooling the suspensions with the suspensions from the first round. The pooled solution was reduced using 4 mM DTT for 30 min at 25 °C shaking at 1000 rpm. The proteins were then alkylated using 10 mM iodoacetamide and incubating for 45 min at 25 °C shaking at 1000 rpm and protected from light. Proteins were then digested with 0.5 µg of trypsin overnight at 25 °C shaking at 700 rpm. The next day proteins were quenched using 40 µL of 10% formic acid and desalted using an Oasis Cartridge. Samples were vacuum-dried and labeled with iTRAQ4 (Sciex) kits; the specific iTRAQ labels used for each pair of bait or control IP replicates are indicated in Supplementary Data 6.

#### Liquid chromatography-tandem mass spectrometry (LC-MS/MS; for “mB.SMC.ADAMTS7” and “mB.SMC.EDN1” datasets)

Reconstituted peptides were separated on an online nanoflow EASY-nLC 1000 UHPLC system (Thermo Scientific) and analyzed on a benchtop Orbitrap Q Exactive Plus mass spectrometer (Thermo Scientific). The peptide samples were injected onto a capillary column (Picofrit with 10 μm tip opening/75 μm diameter, New Objective, PF360-75-10-N-5) packed in-house with 20 cm C18 silica material (1.9 μm ReproSil-Pur C18-AQ medium, Dr. Maisch GmbH, r119.aq). The UHPLC setup was connected with a custom-fit microadapting tee (360 μm, IDEX Health & Science, UH-753), and capillary columns were heated to 50 °C in column heater sleeves (Phoenix-ST) to reduce backpressure during UHPLC separation. Injected peptides were separated at a flow rate of 200 nL/min with a linear 150 min gradient from 94% solvent A (3% acetonitrile, 0.1% formic acid) to 35% solvent B (90% acetonitrile, 0.1% formic acid), followed by a linear 8 min gradient from 35% solvent B to 60% solvent B and a 3 min ramp to 90% B. The Q Exactive instrument was operated in the data-dependent mode acquiring HCD MS/MS scans (*R*=17,500) after each MS1 scan (*R*=70,000) on the 12 most abundant ions using an MS1 ion target of 3 × 10^6^ ions and an MS2 target of 5 × 10^4^ ions. The maximum ion time utilized for the MS/MS scans was 120 ms; the HCD-normalized collision energy was set to 28; the dynamic exclusion time was set to 20 s, and the peptide match and isotope exclusion functions were enabled.

#### Basic reversed-phase (BRP) fractionation followed by LC-MS/MS (for “mB.EC.ARHGEF26” dataset)

To reduce sample complexity, iTRAQ labeled peptide samples were separated by high pH reversed-phase separation as previously described^87^, but scaled down to use a 2.1 mm inner diameter RP Zorbax 300 A Extend-C18 column. All fractions were acidified to a final concentration of 1% formic acid and recombined by pooling every 6th fraction in a step-wise concatenation. Reconstituted peptides from each of the 6 BRP fractions were separated on an online nanoflow EASY-nLC 1000 UHPLC system (Thermo Fisher Scientific) and analyzed on a benchtop Orbitrap Q Exactive plus mass spectrometer (Thermo Fisher Scientific). The ~1 μg peptide samples were injected onto a capillary column (Picofrit with 10-μm tip opening/75 μm diameter, New Objective, PF360-75-10-N-5) packed in-house with 20 cm C18 silica material (1.9 μm ReproSil-Pur C18-AQ medium, Dr. Maisch GmbH, r119.aq). The UHPLC setup was connected with a custom-fit microadapting tee (360 μm, IDEX Health & Science, UH-753), and capillary columns were heated to 50 °C in column heater sleeves (Phoenix-ST) to reduce backpressure during UHPLC separation. Injected peptides were separated at a flow rate of 200 nL/min with a linear 84 min gradient from 94% solvent A (3% acetonitrile, 0.1% formic acid) to 35% solvent B (90% acetonitrile, 0.1% formic acid), followed by a linear 8 min gradient from 35% solvent B to 60% solvent B and a 3 min ramp to 90% B. The Q Exactive instrument was operated in the data-dependent mode acquiring HCD MS/MS scans (*R*=17,500) after each MS1 scan (*R*=70,000) on the 12 top most abundant ions using an MS1 ion target of 3 × 10^6^ ions and an MS2 target of 5 × 10^4^ ions. The maximum ion time utilized for the MS/MS scans was 120 ms; the HCD-normalized collision energy was set to 29; the dynamic exclusion time was set to 20 s, and the peptide match and isotope exclusion functions were enabled.

#### Protein quantification

Mass spectra were analyzed using Spectrum Mill (v7.0; https://proteomics.broadinstitute.org). For peptide identification, MS/MS spectra were searched against the human UniProt database to which a set of common laboratory contaminant proteins was appended. Search parameters included: ESI-QEXACTIVE-HCD scoring parameters, trypsin enzyme specificity with a maximum of two missed cleavages, 40% minimum matched peak intensity, ± 20 ppm precursor mass tolerance, ± 20 ppm product mass tolerance. Carbamidomethylation of cysteines and iTRAQ4 full labeling of lysines and peptide n-termini were set as fixed modifications. Allowed variable modifications were oxidation of methionine (M), acetyl (ProtN-term), and deamidated (N), with a precursor MH+ shift range of −18 to 64 Da. Identities interpreted for individual spectra were automatically designated as valid by optimizing score and delta rank1-rank2 score thresholds separately for each precursor charge state in each LC-MS/MS while allowing a maximum target-decoy-based FDR of 1.0% at the spectrum level. Identified peptides were organized into protein groups and subgroups (isoforms and family members) with Spectrum Mill’s subgroup-specific option enabled, so that peptides shared between subgroups were ignored when using report ion intensities to perform protein-level quantitation.

### IP-MS data processing and analysis

#### Data processing

Starting with the protein-level quantification report for each IP-MS experiment, we performed data processing as follows: (1) log_2_ transformation and median normalization of the protein intensity values in each bait (i.e., index protein) or control IP sample; (2) removal of non-human and uncharacterized proteins, contaminants (e.g., keratins, keratin-associated proteins, trypsins, etc.), unresolved isoforms (i.e., multiple isoforms of the same protein that showed up with identical intensity values in MS), and proteins supported by <2 unique peptides; (3) mapping the remaining proteins to their corresponding HGNC gene symbols and GRCh37/hg19 genomic positions using Ensembl^88^; (4) imputing missing intensity values in each sample by randomly sampling from a normal distribution with a width of 0.3 standard deviation (SD) and downshift of 1.8 SD compared to the observed intensity distribution^16,89^; (5) calculated protein log_2_ fold change (FC) values for each pair of bait vs. control replicate samples.

#### Genoppi analysis

We used the Genoppi R package^16^ (v1.0) to perform QC and analyze each processed IP-MS dataset. Pearson’s correlation of log_2_ FC values between replicates was calculated to assess overall robustness of the IP-MS experiment. Average log_2_ FC, *P* value, and Benjamini-Hochberg false discovery rate (FDR) for each protein were calculated using a one-sample moderated t-test from limma^90^ to identify significant proteins with log_2_ FC >0 and FDR ≤0.1 (i.e., proteins with significantly higher abundance in the bait IPs compared to the controls); these proteins were defined as significant interactors of the index protein in downstream analyses. Using these statistics, we performed QC to identify a subset of high-quality datasets in which the replicate log_2_ FC correlation was >0.6 and the index protein itself was significant at log_2_ FC >0 and FDR ≤0.1, and restricted all subsequent analyses to these datasets. We also assessed the overlap between significant proteins in each dataset and known interactors of the index protein in the InWeb database^17^ (as curated in the Genoppi R package) to distinguish between published vs. potentially novel interactions in our results. Analysis results, experimental details, and summary statistics for the subset of datasets that passed QC are provided in Supplementary Data 5 and 6.

### Comparing IP-MS datasets

#### Across experimental conditions

In order to compare IP-MS datasets generated using different IP methods (endogenous or overexpression/tagging), MS facilities (Broad or Whitehead), or cell types (HAEC or HCASMC), we calculated various QC metrics for each dataset, including: replicate log_2_ FC correlation, number of detected and significant (log_2_ FC >0 and FDR ≤0.1) proteins, number of detected and significant ribosomal proteins (i.e., proteins with RPL- or RPS- prefix in gene symbols), and overlap enrichment between significant proteins and known InWeb interactors. We then performed two-tailed Wilcoxon rank sum tests to assess if the distribution of each metric is significantly different between datasets generated under different conditions.

#### Overlap of interactors

For IP-MS datasets of the same index protein, we used the ggVennDiagram R package (v1.2.2) to visualize the number of interactors that overlap between the datasets. In addition, we performed a one-tailed hypergeometric test to assess the significance of overlap between each pair of IPs using the following definitions: (1) the total “population” (*N*) consists of all genes that were detected in both IPs; (2) the “success in population” (*k*) is the subset of *N* that are significant interactors in IP1; (3) the “sample” (*n*) is the subset of *N* that are significant interactors in IP2; (4) the “success in sample” (*x*) is the overlap between *k* and *n*.

### Defining interactors and non-interactors in the PPI networks

Using the IP-MS analysis results, we defined lists of interactors vs. non-interactors for each index protein to generate combined PPI networks and to perform downstream enrichment analyses. Specifically, the non-interactors were used as background controls in conditional enrichment analyses, in which we aimed to identify significant biology captured by the index protein interactors while accounting for the cell-type-specific nature of our PPI data. For each individual IP-MS dataset, significant proteins with log_2_ FC >0 and FDR ≤0.1 were defined as “interactors” while other detected proteins were defined as “non-interactors”; the index protein used as the bait in the IP was excluded from these lists. When combining results from multiple IP-MS datasets (e.g., all IPs for the same index protein, all IPs performed in the same cell type, etc.), proteins that were significant in ≥1 dataset were defined as “interactors”; proteins that were detected in ≥1 dataset but were not significant in any dataset were defined as “non-interactors”; all index proteins for the source IPs were excluded. Furthermore, for index proteins with IP-MS data in both EC and SMC, we subsetted the data by cell type to define additional networks that contain interactors identified exclusively in EC (EC only), exclusively in SMC (SMC only), in both cell types (Intersect), or in either cell type (Union). Supplementary Data 8 provides additional details on the generated PPI networks, including the summary counts and the full lists of interactors vs. non-interactors in each network.

### Assessing overlap with PPI databases

To further assess whether the identified PPIs have been reported in the literature, we compared them against data from six PPI databases/datasets (Supplementary Data 8). Three datasets are curated and described in the Genoppi R package (v1.0): (1) InWeb^17^; (2) BioPlex^18^ (v3.0, HEK293T); and (3) iRefIndex^19^ (v17.0). The other three datasets are: (4) the HuRI HI-union network, from Supplementary Table 11 of ref. ^20^; (5) the STRING^21^ (v11.5) human physical subnetwork, downloaded from https://string-db.org/cgi/download?sessionId=bpij0JN28bsF; and (6) the PCNet^22^ network, retrieved from the Network Data Exchange (NDEx) with UUID f93f402c-86d4-11e7-a10d-0ac135e8bacf.

### Generating PPI network plots

To visualize the combined PPI networks containing all index proteins and their interactors identified in EC or SMC, we used the igraph (v1.2.5) and qgraph (v1.6.5) R packages to generate undirected network graphs, in which a vertex represents an index or interactor protein and an edge represents a significant index protein-interactor interaction observed in our IP-MS data.

### Tissue and gene set enrichment analysis

We performed one-tailed hypergeometric tests to assess the significance of overlap between the interactors in our PPI networks and various gene sets. The gene sets we tested have all been curated in the Genoppi R package (v1.0) and include: (1) tissue-specific gene sets defined using GTEx RNA-seq data^40^; (2) tissue-specific gene sets defined using GTEx proteomic data^44^; (3) MSigDB Hallmark and Reactome gene sets^45,46^; and (4) GO BP, CC, and MF terms^47,48^. To assess GTEx tissue enrichment using RNA or proteomic data, we first performed a global enrichment analysis between each tissue-specific gene set and each PPI network using the following definitions: (1) the total “population” (*N*) consists of all genes that have been annotated in ≥1 gene sets; (2) the “success in population” (*k*) is the subset of *N* that are interactors in the PPI network; (3) the “sample” (*n*) is the subset of *N* that are in the current gene set; (4) the “success in sample” (*x*) is the overlap between *k* and *n*. As comparison, we also performed the analogous global analysis for the non-interactors linked to each network, as well as a conditional analysis in which we compared the interactors against the non-interactors (i.e., by further restricting the “population” defined above to genes encoded by interactors or non-interactors in the network). For the MSigDB and GO analyses, we performed analogous conditional tests that compared the interactors against the non-interactors, to identify gene sets that are significantly enriched even when accounting for the background cellular context of our data.

### Whole proteome analysis

Protein expression values were derived from the whole proteome dataset in Supplementary Data 7 of ref. ^49^, which analyzed cardiac fibroblasts (CF), endothelial cells (EC), and smooth muscle cells (SMC) collected during cardiovascular surgery and adipose fibroblasts (AF) as a control cell type. We compared the expression of index protein interactors, non-interactors, and other proteins found in the whole proteome dataset using two-tailed Wilcoxon rank sum tests.

### Genetic risk enrichment analysis

We used MAGMA^50^ (v1.09) and CAD GWAS summary statistics from a meta-analysis of the UK Biobank and CARDIoGRAMplusC4D^51^ to assess whether the interactor genes in our PPI networks are enriched for polygenic risk of CAD. We also performed analogous MAGMA analyses using GWAS summary statistics of aortic size^56^ (ascending aortic (AA) or descending aortic (DA) diameter) and stroke^57^ (any stroke (AS), any ischemic stroke (AIS), large-artery atherosclerotic stroke (LAS), cardioembolic stroke (CES), or small-vessel stroke (SVS)). First, we annotated protein-coding genes in the Ensembl^88^ GRCh37 database with variants in the 1000 Genomes^91^ (phase 3) EUR panel using a flanking window of ± 50 kb; variants in the major histocompatibility complex region (chr6:28.5M–33.4M) were excluded due to its complex LD structure. Next, for each GWAS dataset, gene-based *P* values were calculated using the SNP-wise Mean model and the 1000 Genomes EUR panel. Then, for each GWAS dataset and each PPI network, the gene set analysis model was used to compare the interactor genes in the network against the rest of the genome (for the global enrichment tests) or the non-interactor genes (for the conditional enrichment tests), computing a one-tailed *P* value that indicates whether the interactors are more strongly associated with the GWAS phenotype.

### Using PPI networks to prioritize additional CAD-risk genes from GWAS data

Starting with 157 genome-wide significant index variants reported in the UK Biobank and CARDIoGRAMplusC4D GWAS^51^, we used PLINK^92^ (v1.9) and the 1000 Genomes^91^ (phase 3) EUR panel to define LD locus boundaries for each variant, which span SNPs with *r*^2^ >0.6 ± 50 kb on either end. Next, we used gene annotations from Ensembl^88^ to extract all protein-coding genes overlapping the LD loci and intersected them with index genes and interactors derived from our IP-MS data. We plotted the resulting list of prioritized genes in a “social Manhattan plot” where the chromosomal position of each gene is shown on the *x* axis and the GWAS *P* value of its tagging SNP is shown on the *y* axis, while the edges connecting the genes represent observed protein interactions between them.

### Statistics and reproducibility

The genetic risk enrichment analysis was performed using MAGMA (v1.09). Other statistical analyses were performed in R. Analysis scripts with package and version documentation are deposited at GitHub (https://github.com/lagelab/CAD_PPI). Statistical tests and significance cutoffs used are described in “Methods” and figure legends.

The IP-MS experimental replicates are described in “Methods” under “Co-immunoprecipitation using index proteins as baits”. Briefly, for each experiment, each bait or control IP mixture was split into three replicates. Two of the three replicates were submitted for mass spectrometry and the remaining replicate was used for quality control by western blot analyses (Supplementary Figs. 1 and 2).

### Reporting summary

Further information on research design is available in the Nature Portfolio Reporting Summary linked to this article.

### Supplementary information

Supplementary Information

Description of Additional Supplementary Files

Supplementary Data 1

Supplementary Data 2–15

Reporting Summary

## Data Availability

The mass spectra from IP-MS experiments and the protein sequence databases used for searches have been deposited at MassIVE (https://massive.ucsd.edu) with identifiers MSV000091373 (data from Whitehead Proteomics Core Facility) and MSV000091699 (data from Broad Proteomics Platform). Source data for figures are documented in Supplementary Data 1.

## Code availability

Original code has been deposited at GitHub (https://github.com/lagelab/CAD_PPI) and Zenodo (10.5281/zenodo.8415025).
