# Supplementary material for: Protein interaction networks in the vasculature prioritize genes and pathways underlying coronary artery disease
Source: Commun Biol. 2024 Jan 12;7:87. doi: 10.1038/s42003-023-05705-1 (PMC10786878; doi:10.1038/s42003-023-05705-1)
Supplement: Supplementary file 1 — Supplementary Information [file 42003_2023_5705_MOESM1_ESM.pdf]

Supplementary Information for:
Protein interaction networks in the vasculature prioritize genes and pathways underlying coronary artery disease

This PDF file includes:

Supplementary Figures 1 to 19

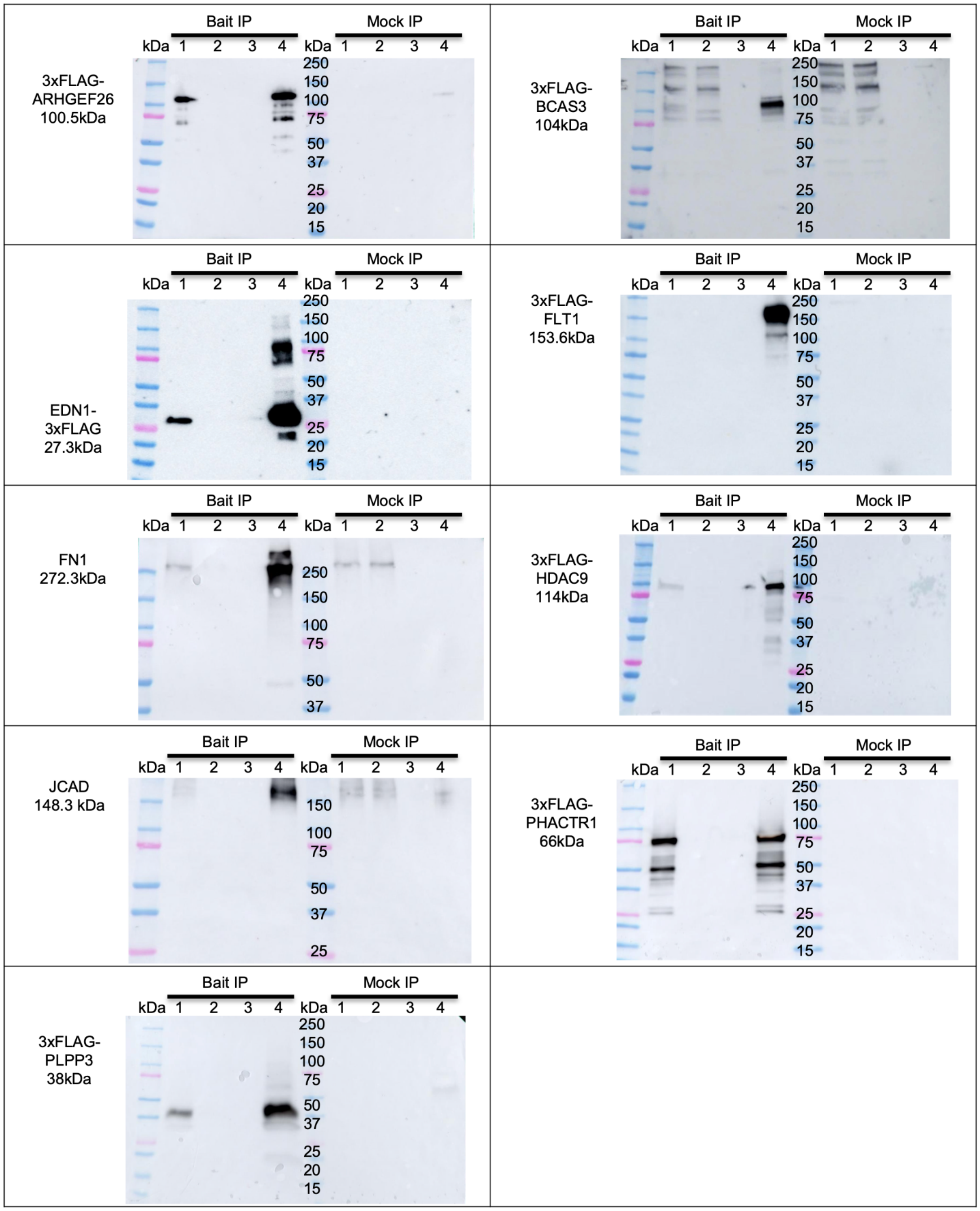

Supplementary Figure 1. QC Western blots for IP samples submitted for MS analyses in EC. An independent IP was performed in parallel with the IP samples used for MS, and subjected to Western Blot analyses for each bait. Lane #1, total input lysate; Lane #2, post-IP flow through of lysate; Lane #3, buffer from first post-IP wash (to detect potential bait loss during washing); Lane #4, elution from washed beads after IP. Note the significant enrichment of bait proteins detected in all bait IP, but little or none in mock IP. Each blot represents one of 3 IP replicates.

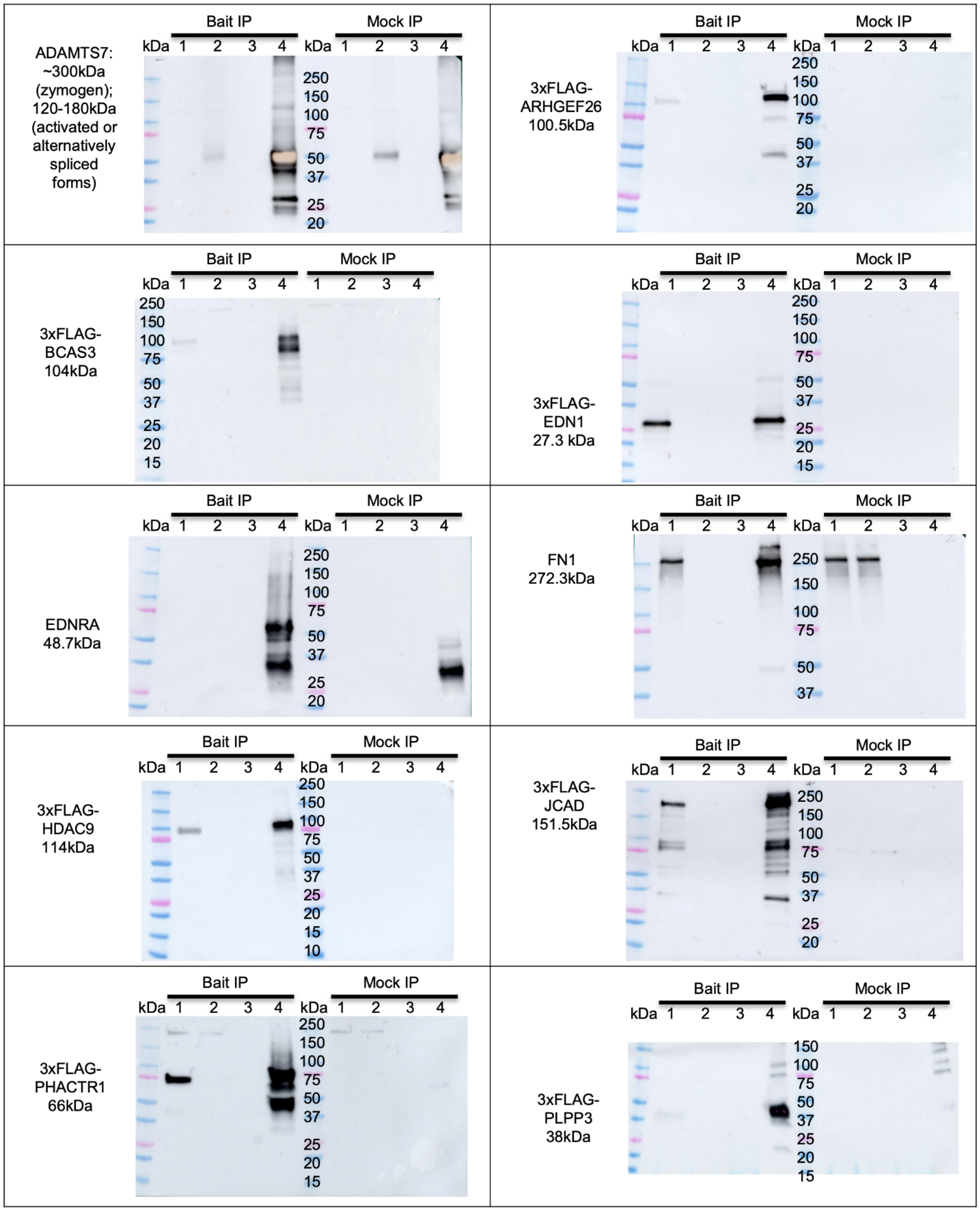

Supplementary Figure 2. QC Western blots for IP samples submitted for MS analyses in SMC. An independent IP was performed in parallel with the IP samples used for MS, and subjected to Western Blot analyses for each bait. Lane #1, total input lysate; Lane #2, post-IP flow through of lysate; Lane #3, buffer from first post-IP wash (to detect potential bait loss during washing); Lane #4, elution from washed beads after IP. Note the significant enrichment of bait proteins detected in all bait IP, but little or none in mock IP. Each blot represents one of 3 IP replicates.

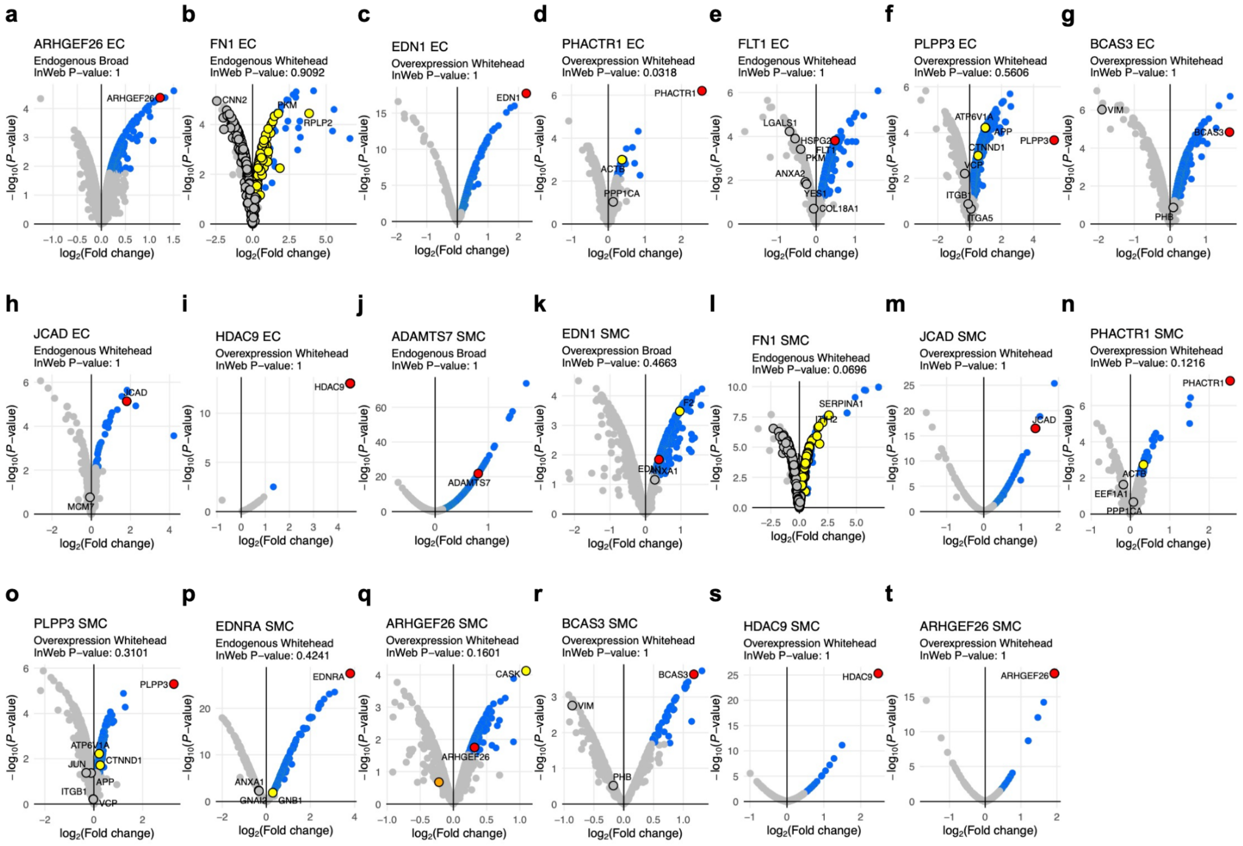

Supplementary Figure 3. Volcano plots of the 20 IP-MS datasets that passed QC. The bait protein, cell type (EC or SMC), IP method (endogenous or overexpression), and MS facility (Broad or Whitehead) used to generate each dataset are indicated at the top of each panel. In each volcano plot, the bait protein, its significant interactors ($\log_2$ FC > 0 and FDR ≤ 0.1), and other non-significant proteins (i.e., non-interactors) are shown in red, blue, and gray, respectively. Statistics were calculated using two IP-MS replicates. Known interactors of the bait in the InWeb database are marked by black border circles, with those that are significant in our data additionally highlighted in yellow; hypergeometric p-value indicating overlap enrichment between significant interactors and known InWeb interactors is shown above each plot.

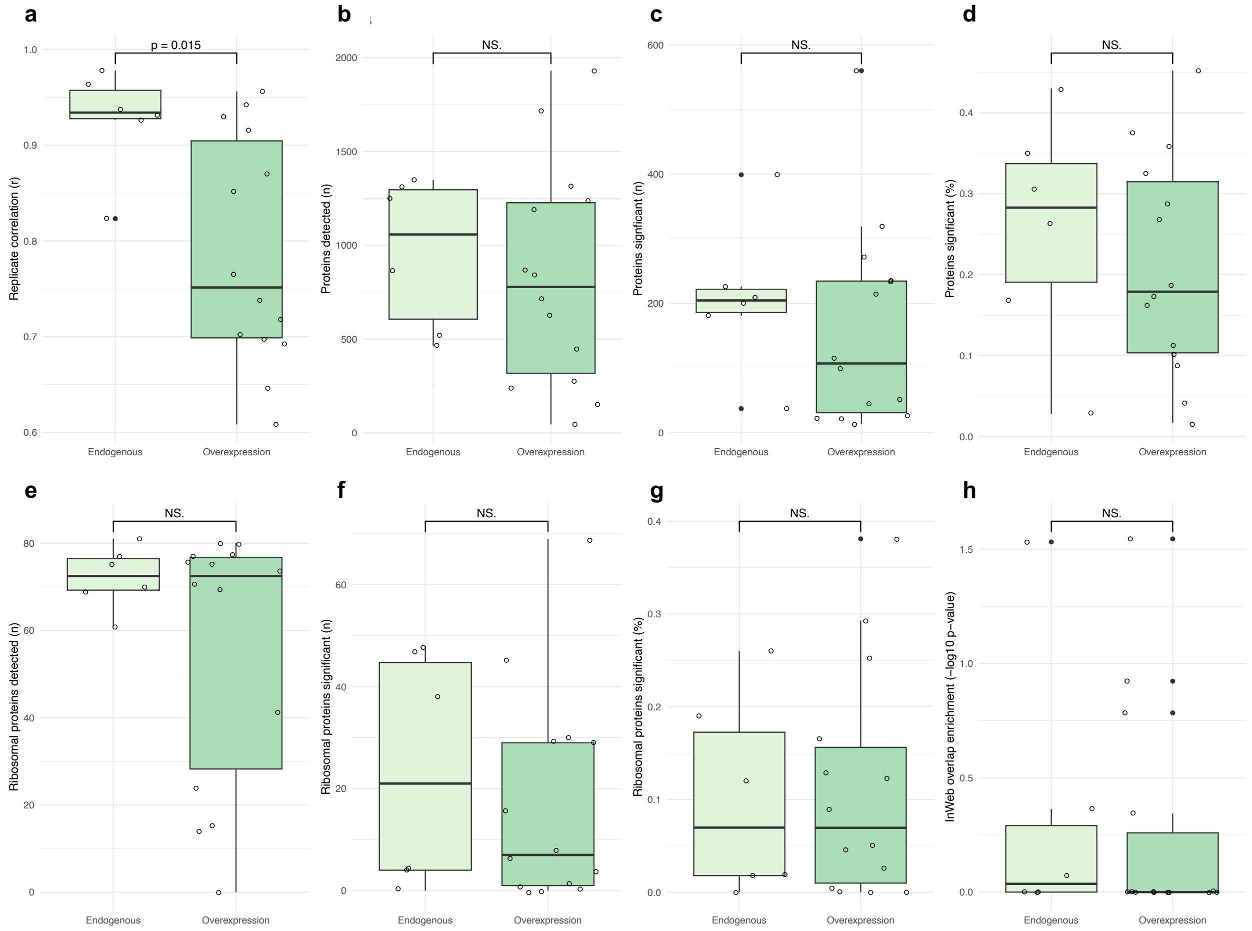

Supplementary Figure 4. Comparison of QC'ed IP-MS datasets generated from endogenous vs. overexpression (i.e., flag-tagged) IPs. Each panel contains box plots comparing a different QC metric: **(a)** Pearson's correlation of $\log_2$ fold change (FC) between replicates; **(b)** number of detected proteins; **(c)** number of significant proteins (with $\log_2$ FC > 0 and FDR ≤ 0.1); **(d)** percentage of significant proteins (out of detected proteins); **(e)** number of detected ribosomal proteins (i.e., RPL or RPS proteins); **(f)** number of significant ribosomal proteins; **(g)** percentage of significant ribosomal proteins (out of significant proteins); **(h)** $-\log_{10}$ hypergeometric *p*-value indicating overlap enrichment between significant proteins and known interactors found in the InWeb database. Data points representing the individual IP datasets (n=20) are overlaid on top of the box plots; center line, median; box limits, upper and lower quartiles; whiskers, 1.5x interquartile range. A two-tailed Wilcoxon rank sum test was performed to compare the endogenous vs. overexpression IP distributions for each QC metric; the resulting *p*-values or "NS." are shown above the corresponding box plots for nominally significant (i.e., *P* < 0.05) or non-significant comparisons, respectively.

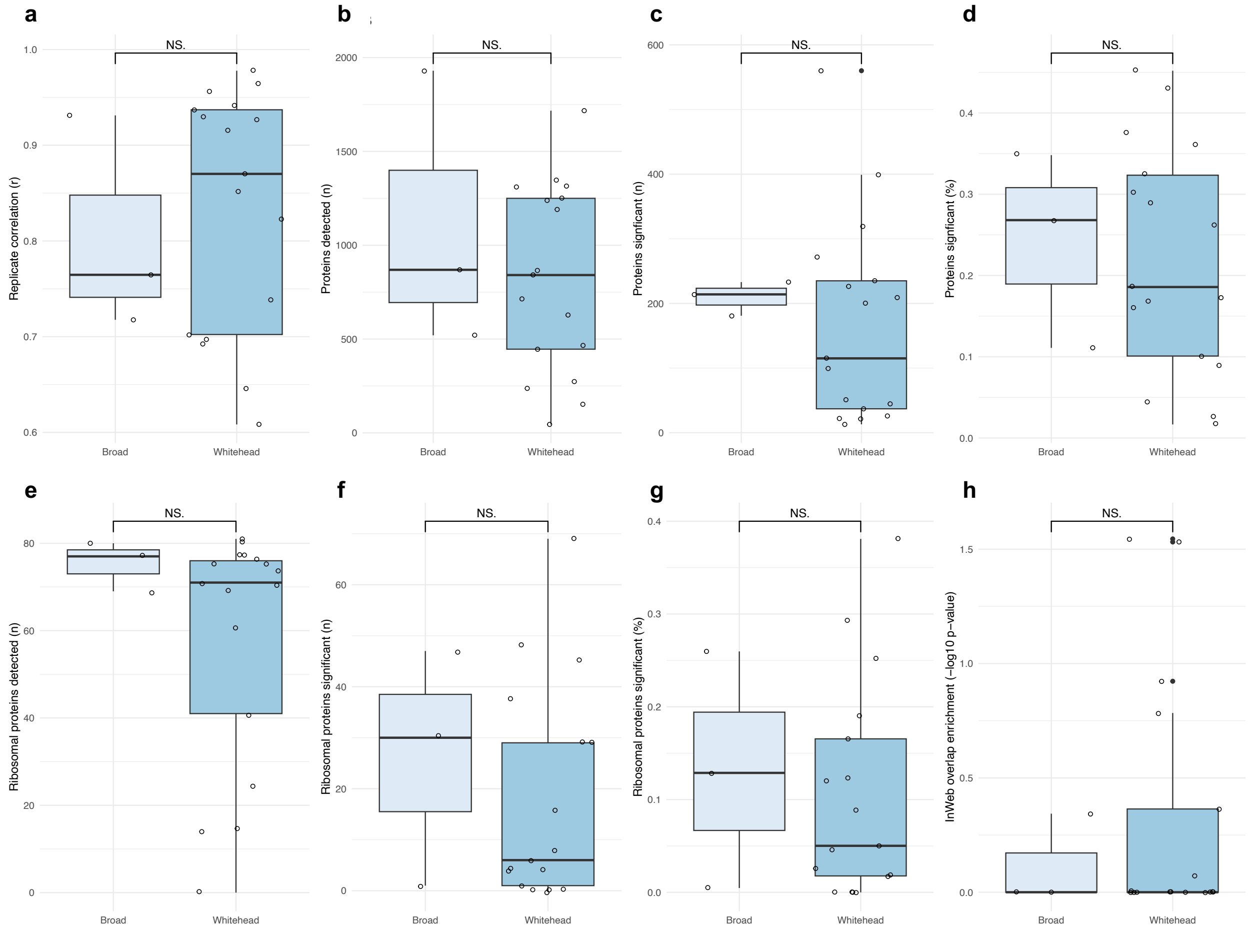

Supplementary Figure 5. Comparison of QC'ed IP-MS datasets generated by the Broad vs. Whitehead facilities. Each panel contains box plots comparing a different QC metric: **(a)** Pearson's correlation of $\log_2$ fold change (FC) between replicates; **(b)** number of detected proteins; **(c)** number of significant proteins (with $\log_2$ FC > 0 and FDR ≤ 0.1); **(d)** percentage of significant proteins (out of detected proteins); **(e)** number of detected ribosomal proteins (i.e., RPL or RPS proteins); **(f)** number of significant ribosomal proteins; **(g)** percentage of significant ribosomal proteins (out of significant proteins); **(h)** $-\log_{10}$ hypergeometric *p*-value indicating overlap enrichment between significant proteins and known interactors found in the InWeb database. Data points representing the individual IP datasets (n=20) are overlaid on top of the box plots; center line, median; box limits, upper and lower quartiles; whiskers, 1.5x interquartile range. A two-tailed Wilcoxon rank sum test was performed to compare the Broad vs. Whitehead IP distributions for each QC metric; the resulting *p*-values or "NS." are shown above the corresponding box plots for nominally significant (i.e., $P < 0.05$) or non-significant comparisons, respectively.

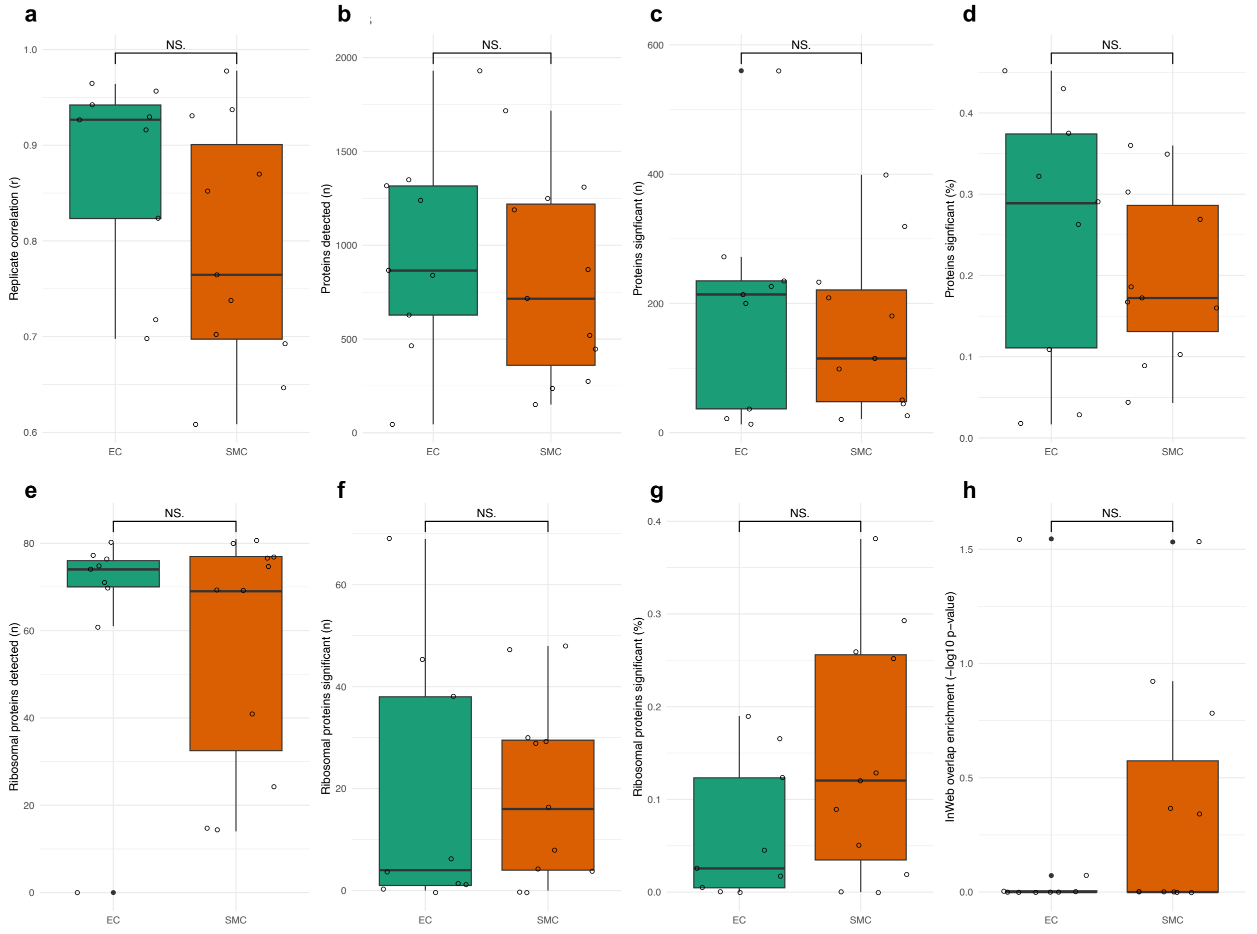

Supplementary Figure 6. Comparison of QC'ed IP-MS datasets derived from EC or SMC. Each panel contains box plots comparing a different QC metric: **(a)** Pearson's correlation of $\log_2$ fold change (FC) between replicates; **(b)** number of detected proteins; **(c)** number of significant proteins (with $\log_2$ FC > 0 and FDR ≤ 0.1); **(d)** percentage of significant proteins (out of detected proteins); **(e)** number of detected ribosomal proteins (i.e., RPL or RPS proteins); **(f)** number of significant ribosomal proteins; **(g)** percentage of significant ribosomal proteins (out of significant proteins); **(h)** $-\log_{10}$ hypergeometric *p*-value indicating overlap enrichment between significant proteins and known interactors found in the InWeb database. Data points representing the individual IP datasets (n=20) are overlaid on top of the box plots; center line, median; box limits, upper and lower quartiles; whiskers, 1.5x interquartile range. A two-tailed Wilcoxon rank sum test was performed to compare the EC vs. SMC IP distributions for each QC metric; the resulting *p*-values or "NS." are shown above the corresponding box plots for nominally significant (i.e., $P < 0.05$) or non-significant comparisons, respectively.

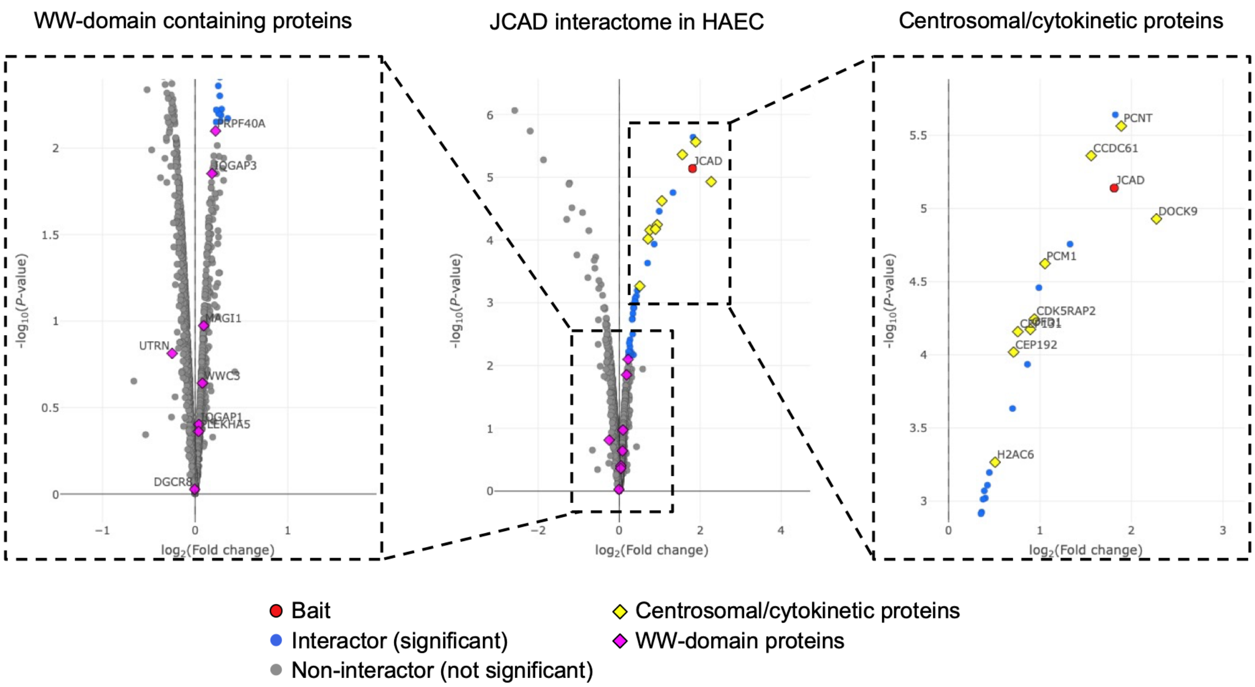

Supplementary Figure 7. Volcano plot showing IP-MS data for JCAD in EC. The bait protein (JCAD), its significant interactors ($\log_2$ FC > 0 and FDR ≤ 0.1), and other non-significant proteins (i.e., non-interactors) are shown in red, blue, and gray, respectively. Statistics were calculated using two IP-MS replicates. WW-domain-containing proteins are highlighted by pink diamonds (left box); centrosomal proteins or proteins with known roles in cytokinesis are highlighted by yellow diamonds (right box).

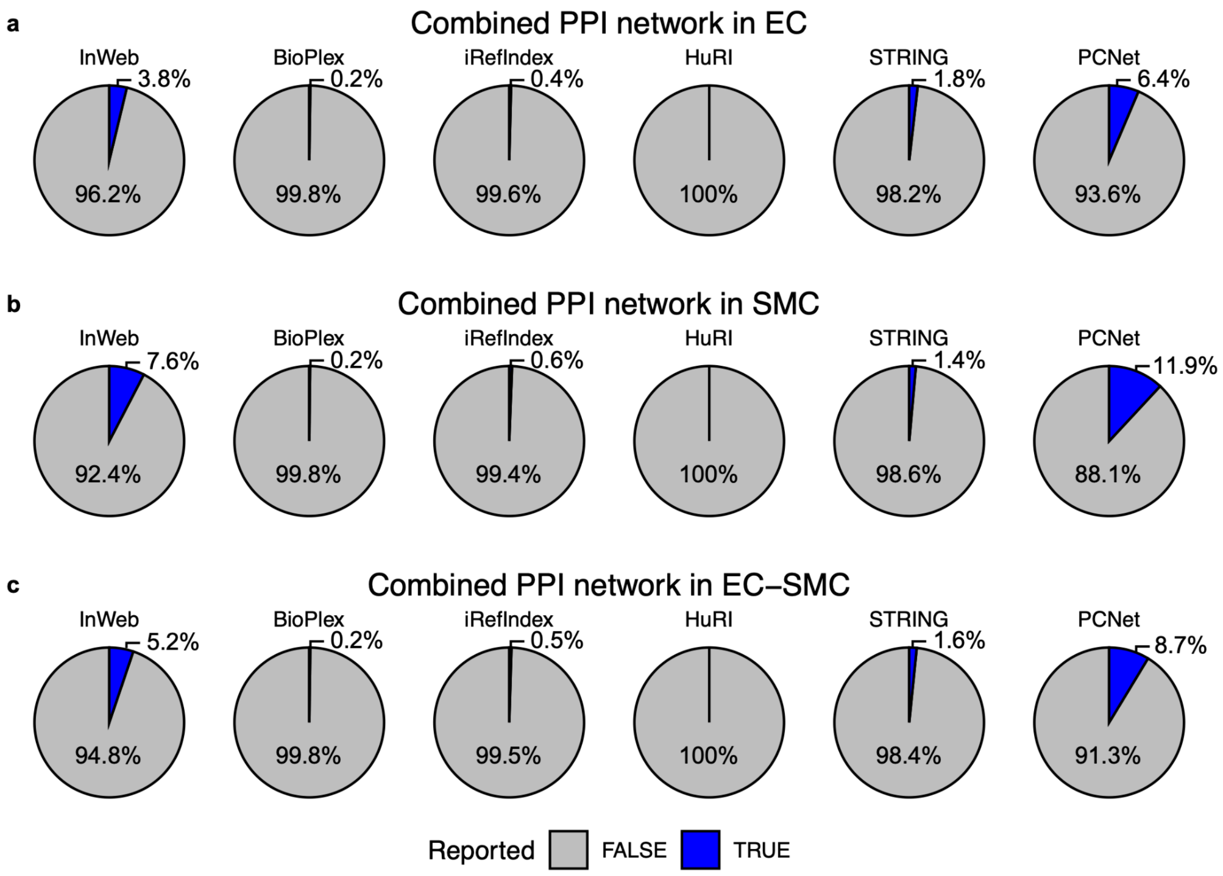

Supplementary Figure 8. Overlap between PPIs identified in this study vs. external PPI databases. Pie charts showing the percentage of interactions in EC **(a)**, SMC **(b)**, or either cell type **(c)** that have been reported in six PPI databases (named above pie charts). Only physical interactions are included in InWeb, BioPlex, iRefIndex, HuRI, and STRING (physical subnetwork); both physical and functional interactions are included in PCNet.

a **Endothelial cell**

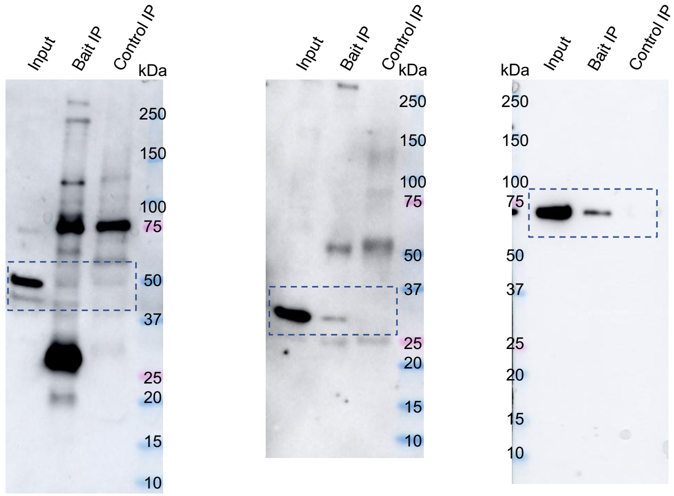

IP	EDN1	PLPP3	EDN1
**Detection**	PDIA6 (48k Da)	RPL7A (30 kDa)	HSPA9 (75 kDa)

b **Smooth muscle cell**

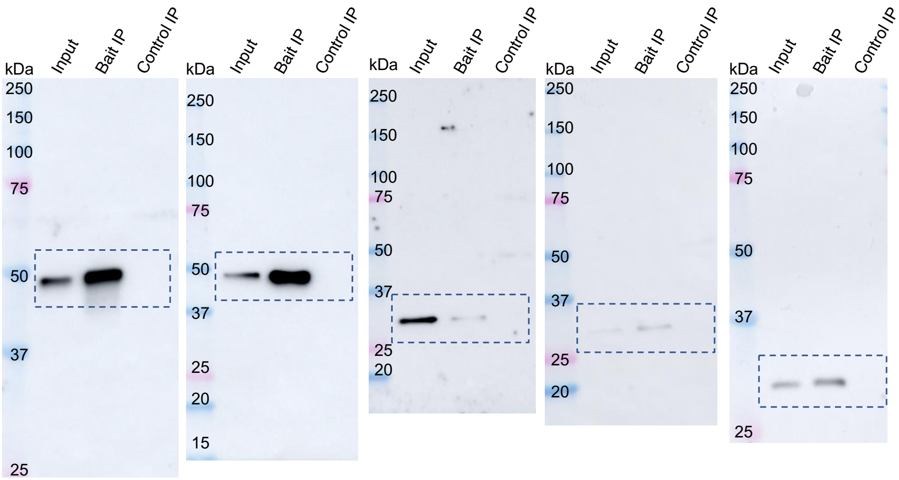

IP	JCAD	PLPP3	EDN1	FN1	JCAD
**Detection**	PDIA6 (48 kDa)	PDIA6 (48 kDa)	RPL7A (30 kDa)	RPL7A (30 kDa)	RPL7A (30 kDa)

Supplementary Figure 9. Validation of selected recurrent interactors (PDIA6, RPL7A, HSPA9) identified in both EC and SMC. IPs of the index proteins were performed in EC **(a)** or SMC **(b)**, then subjected to Western blot analysis to detect the presence of selected interactors. Control IPs for FLAG-tagged baits were performed using an anti-FLAG antibody in empty-vector-transfected cells; control IPs for endogenous baits were performed using a non-specific rabbit or mouse IgG antibody. Antibodies used in IP and Western blot are listed in **Supplementary Data 6** and **Methods**. Each blot represents one of two IP replicates. Bands corresponding to the expected molecular weight of the Detection protein (shown in parentheses) are marked by dashed boxes.

a

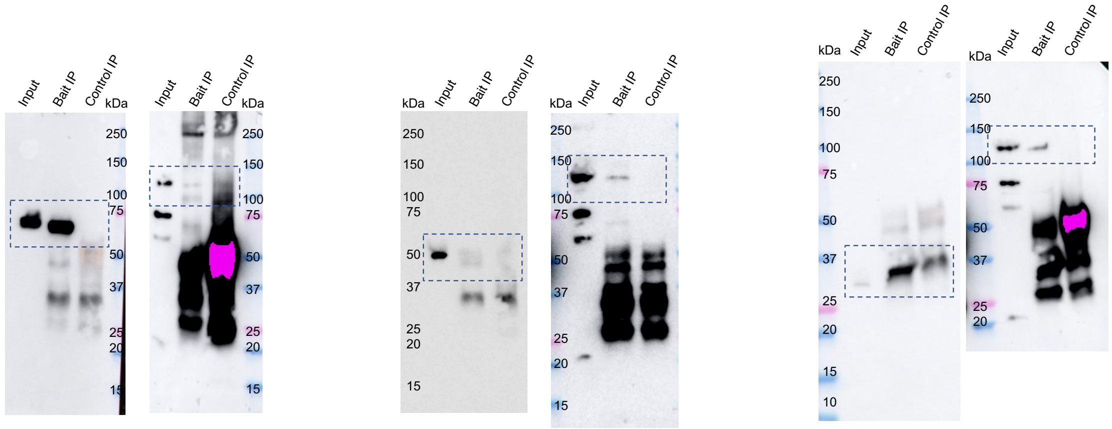

IP	IGF2BP1	IGF2BP1
**Detection**	IGF2BP1 (63 kDa)	ADAMTS7 (120-180 kDa)

IP	PDIA6	PDIA6
**Detection**	PDIA6 (48 kDa)	ADAMTS7 (120-180 kDa)

IP	RPL7A	RPL7A
**Detection**	RPL7A (30 kDa)	ADAMTS7 (120-180 kDa)

b

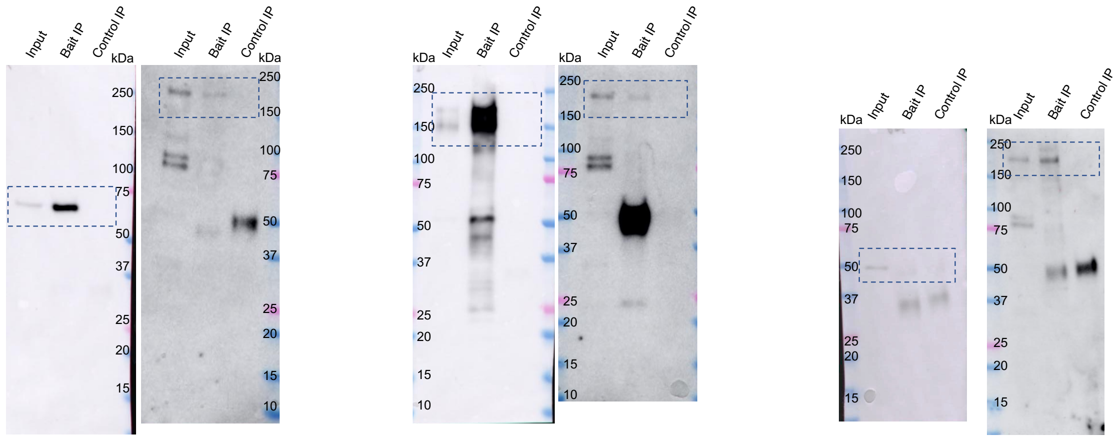

IP	IGF2BP1	IGF2BP1
**Detection**	IGF2BP1 (63 kDa)	JCAD (151.5 kDa)

IP	MAP4	MAP4
**Detection**	MAP4 (210-240 kDa)	JCAD (151.5 kDa)

IP	PDIA6	PDIA6
**Detection**	PDIA6 (48 kDa)	JCAD (151.5 kDa)

Supplementary Figure 10. Validation of selected ADAMTS7 and JCAD interactors in SMC using reciprocal IPs. Individual IPs of ADAMTS7 **(a)** or JCAD **(b)** interactors were performed in SMC, then subjected to Western blot analysis to detect the presence of the respective index protein. Control IPs were performed using a non-specific rabbit IgG antibody. Antibodies used in IP and Western blot are listed in **Methods**. Each blot represents one of two IP replicates. Bands corresponding to the expected molecular weight of the Detection protein (shown in parentheses) are marked by dashed boxes.

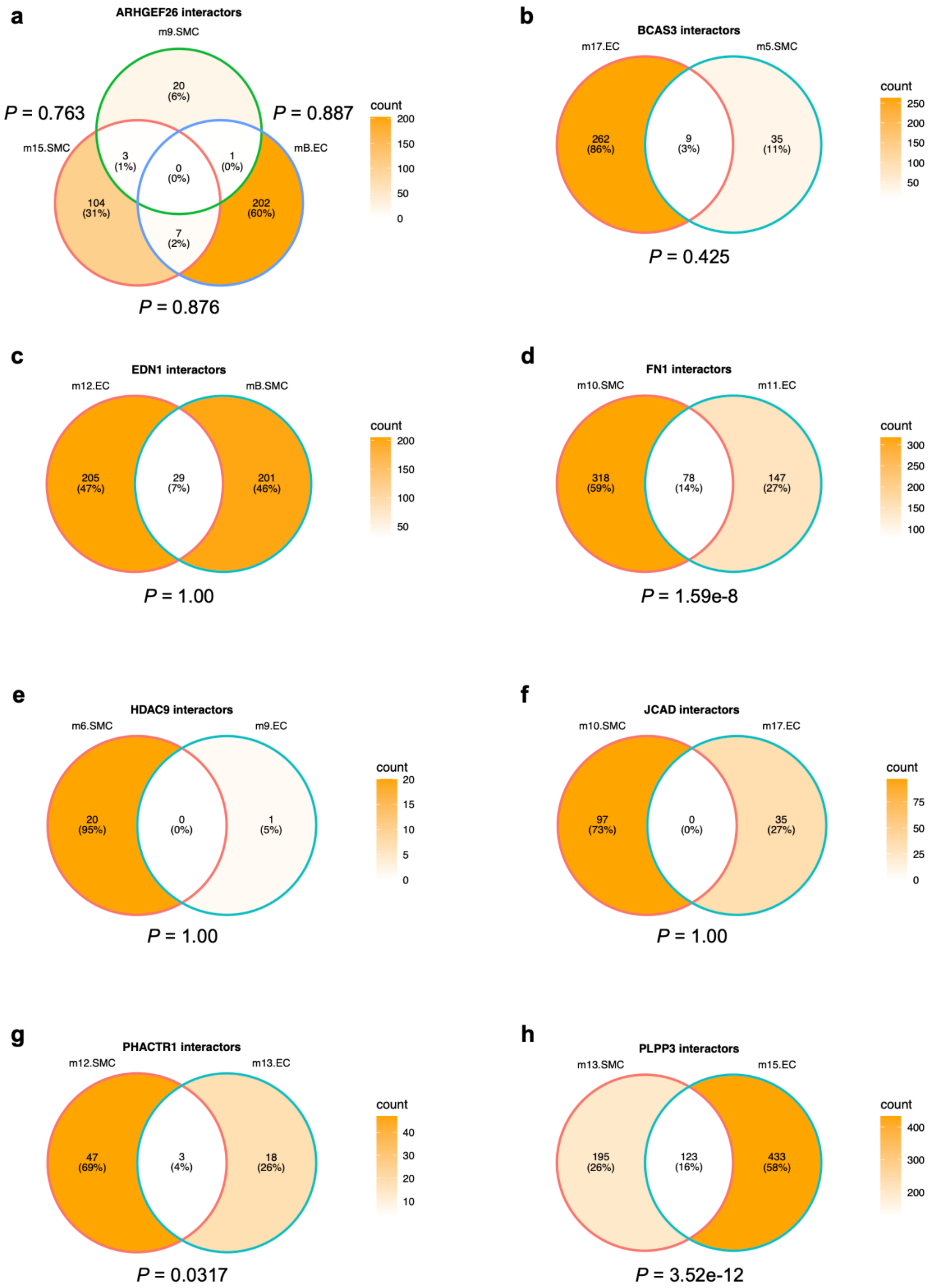

Supplementary Figure 11. Overlap of interactors between IP-MS datasets for the same bait protein. Name of each IP dataset is shown above the Venn diagram and corresponds to the prefix used in **Supplementary Data 5-6**. Overlap enrichment p-value between each pair of IPs was calculated from a one-tailed hypergeometric test; detailed statistics are provided in **Supplementary Data 9**.

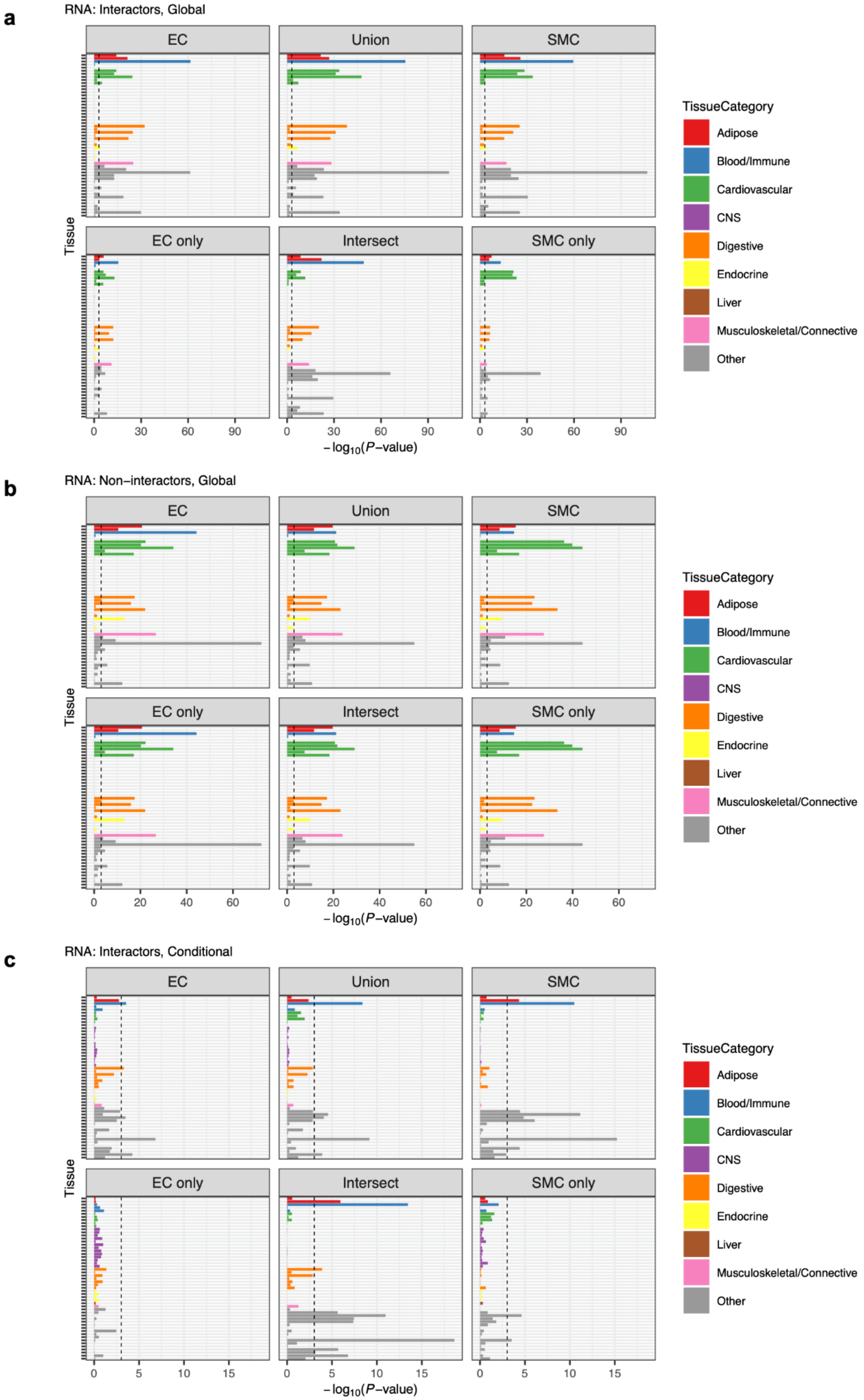
a
RNA: Interactors, Global
EC
Union
SMC
EC only
Intersect
SMC only
Tissue
0
30
60
90
−log10(P−value)
TissueCategory
Adipose
Blood/Immune
Cardiovascular
CNS
Digestive
Endocrine
Liver
Musculoskeletal/Connective
Other
b
RNA: Non−interactors, Global
0
20
40
60
c
RNA: Interactors, Conditional
0
5
10
15

Supplementary Figure 12. Tissue enrichment of the PPI networks calculated using GTEx tissue-specific genes based on RNA-seq data. One-tailed hypergeometric tests were used to calculate the tissue enrichment of index protein interactors compared to a global background **(a)**, non-interactors compared to a global background **(b)**, or index protein interactors compared to a conditional (i.e., non-interactors) background **(c)**. Facets indicate interactors (or non-interactors) identified in EC, SMC, either cell type (Union), both cell types (Intersect), exclusively in EC (EC only), or exclusively in SMC (SMC only). Vertical dashed line indicates $P <$ 0.05/number of tissues. Gene counts used for analysis are shown in **Supplementary Data 10**.

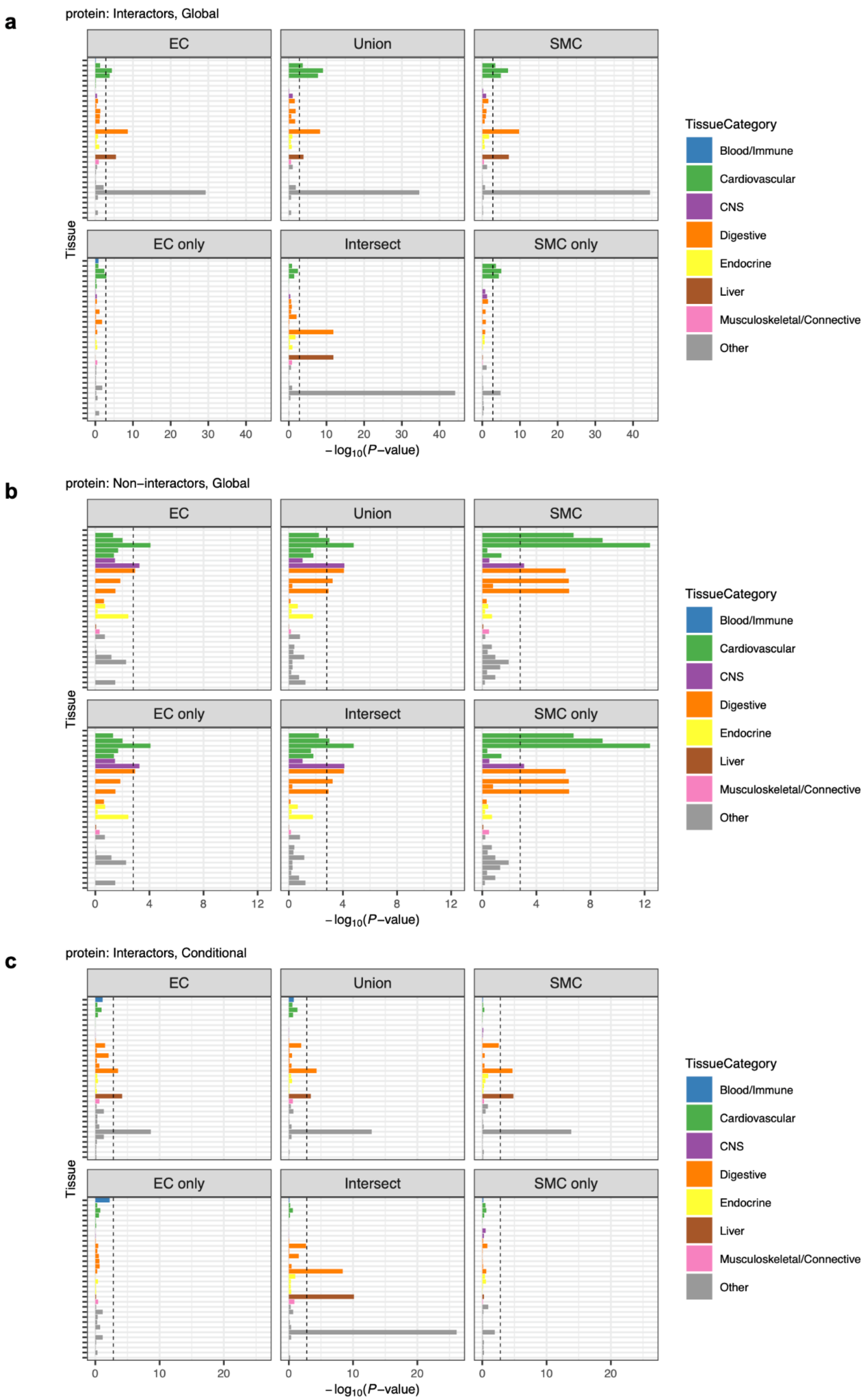
a
protein: Interactors, Global
EC
Union
SMC
EC only
Intersect
SMC only
Tissue
0
10
20
30
40
$-\log_{10}(P\text{-value})$
TissueCategory
Blood/Immune
Cardiovascular
CNS
Digestive
Endocrine
Liver
Musculoskeletal/Connective
Other
b
protein: Non−interactors, Global
EC
Union
SMC
EC only
Intersect
SMC only
Tissue
0
4
8
12
$-\log_{10}(P\text{-value})$
TissueCategory
Blood/Immune
Cardiovascular
CNS
Digestive
Endocrine
Liver
Musculoskeletal/Connective
Other
c
protein: Interactors, Conditional
EC
Union
SMC
EC only
Intersect
SMC only
Tissue
0
10
20
$-\log_{10}(P\text{-value})$
TissueCategory
Blood/Immune
Cardiovascular
CNS
Digestive
Endocrine
Liver
Musculoskeletal/Connective
Other

Supplementary Figure 13. Tissue enrichment of the PPI networks calculated using GTEx tissue-specific genes based on proteomic data. One-tailed hypergeometric tests were used to calculate the tissue enrichment of index protein interactors compared to a global background **(a)**, non-interactors compared to a global background **(b)**, or index protein interactors compared to a conditional (i.e., non-interactors) background **(c)**. Facets indicate interactors (or non-interactors) identified in EC, SMC, either cell type (Union), both cell types (Intersect), exclusively in EC (EC only), or exclusively in SMC (SMC only). Vertical dashed line indicates $P < 0.05/\text{number of tissues}$. Gene counts used for analysis are shown in **Supplementary Data 10**.

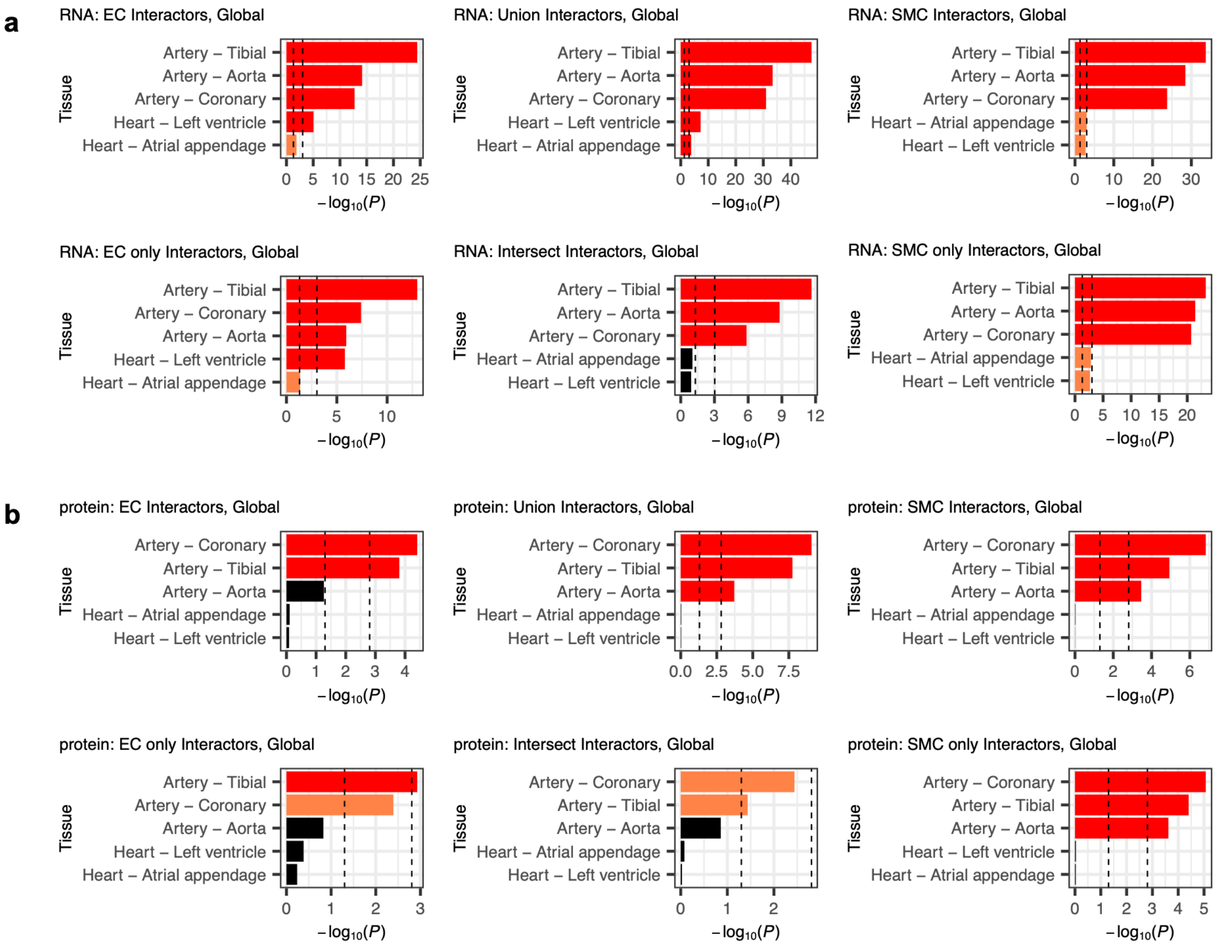

Supplementary Figure 14. Cardiovascular tissue enrichment of the PPI networks calculated using GTEx tissue-specific genes based on RNA-seq (a) or proteomic (b) data. One-tailed hypergeometric tests were used to calculate the tissue enrichment of index protein interactors identified in EC, SMC, either cell type (Union), both cell types (Intersect), exclusively in EC (EC only), or exclusively in SMC (SMC only) compared to a global background. Left and right vertical dashed lines indicate $P < 0.05$ and $P < 0.05$/number of tissues, respectively. Gene counts used for analysis are shown in **Supplementary Data 10**.

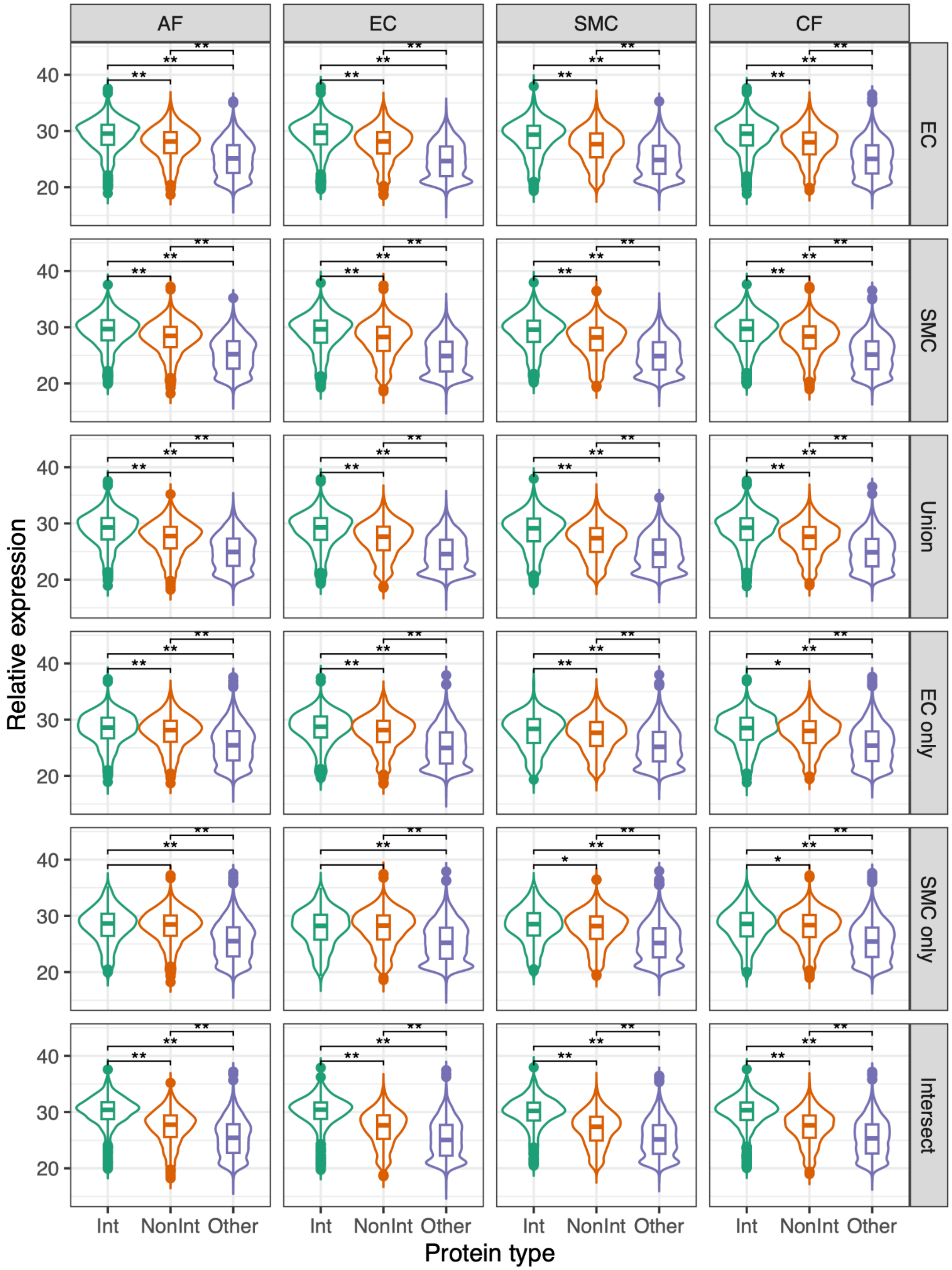
AF
EC
SMC
CF
EC
SMC
Union
EC only
SMC only
Intersect
Relative expression
40
30
20
Int
NonInt
Other
Protein type

Supplementary Figure 15. Protein expression of index proteins interactors and non-interactors in human heart cell types. Expression values were derived from a whole proteome dataset in Doll *et al.*, which analyzed 4 cell types (column facets) including endothelial cells (EC), smooth muscle cells (SMC), and cardiac fibroblasts (CF) harvested during cardiovascular surgery, and adipose fibroblasts (AF) as a control cell type. Expression of index protein interactors (Int) and non-interactors (NonInt) were compared with each other and with other proteins (Other) found in the whole proteome dataset. Row facets indicate interactors and non-interactors identified in EC, SMC, either cell type (Union), both cell types (Intersect), exclusively in EC (EC only), or exclusively in SMC (SMC only). Violin plots show the density distribution of individual data points; center line, box limits, and whiskers represent the median, upper and lower quartiles, and 1.5x interquartile range, respectively. Single and double asterisks indicate $P < 0.05$ and $P < 0.001$ in a two-tailed Wilcoxon rank sum test, respectively.

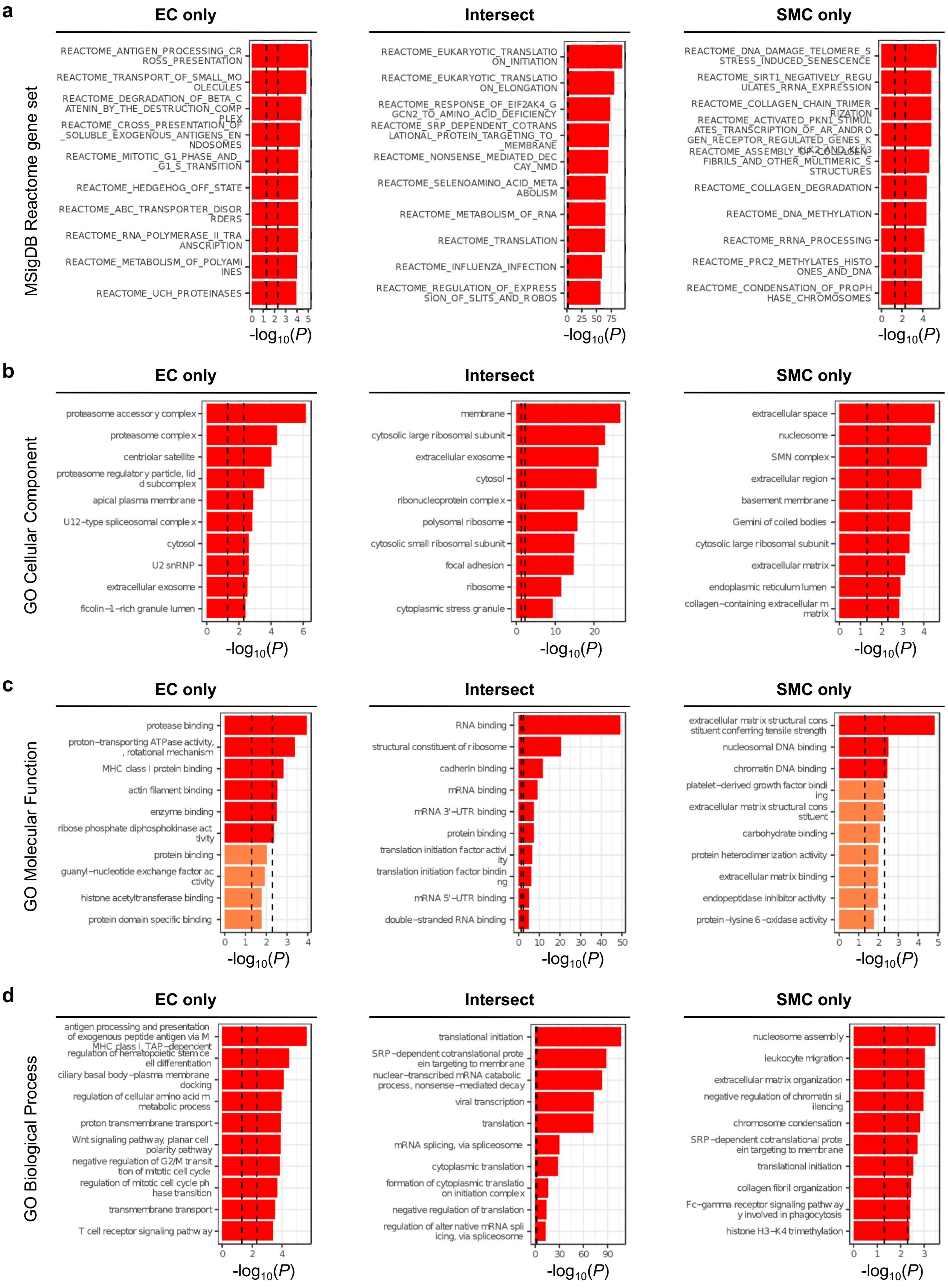
a
EC only
Intersect
SMC only
MSigDB Reactome gene set
REACTOME_ANTIGEN_PROCESSING_CROSS_PRESENTATION
REACTOME_TRANSPORT_OF_SMALL_MOLECULES
REACTOME_DEGRADATION_OF_BETA_CATENIN_BY_THE_DESTRUCTION_COMPLEX
REACTOME_CROSS_PRESENTATION_OF_SOLUBLE_EXOGENOUS_ANTIGENS_ENDOSOMES
REACTOME_MITOTIC_G1_PHASE_AND_G1_S_TRANSITION
REACTOME_HEDGEHOG_OFF_STATE
REACTOME_ABC_TRANSPORTER_DISORDERS
REACTOME_RNA_POLYMERASE_II_TRANSCRIPTION
REACTOME_METABOLISM_OF_POLYAMINES
REACTOME_UCH_PROTEINASES
REACTOME_EUKARYOTIC_TRANSLATION_INITIATION
REACTOME_EUKARYOTIC_TRANSLATION_ELONGATION
REACTOME_RESPONSE_OF_EIF2AK4_GCN2_TO_AMINO_ACID_DEFICIENCY
REACTOME_SRP_DEPENDENT_COTRANSLATIONAL_PROTEIN_TARGETING_TO_MEMBRANE
REACTOME_NONSENSE_MEDIATED_DECAY_NMD
REACTOME_SELENOAMINO_ACID_METABOLISM
REACTOME_METABOLISM_OF_RNA
REACTOME_TRANSLATION
REACTOME_INFLUENZA_INFECTION
REACTOME_REGULATION_OF_EXPRESSION_OF_SLITS_AND_ROBOS
REACTOME_DNA_DAMAGE_TELOMERE_STRESS_INDUCED_SENESCENCE
REACTOME_SIRT1_NEGATIVELY_REGULATES_RRNA_EXPRESSION
REACTOME_COLLAGEN_CHAIN_TRIMERIZATION
REACTOME_ACTIVATED_PKN1_STIMULATES_TRANSCRIPTION_OF_AR_ANDROGEN_RECEPTOR_REGULATED_GENES_KLK2_AND_KLK3
REACTOME_ASSEMBLY_OF_COLLAGEN_FIBRILS_AND_OTHER_MULTIMERIC_STRUCTURES
REACTOME_COLLAGEN_DEGRADATION
REACTOME_DNA_METHYLATION
REACTOME_RRNA_PROCESSING
REACTOME_PRC2_METHYLATES_HISTONES_AND_DNA
REACTOME_CONDENSATION_OF_PROPHASE_CHROMOSOMES
-log10(P)
b
GO Cellular Component
proteasome accessory complex
proteasome complex
centriolar satellite
proteasome regulatory particle, lid subcomplex
apical plasma membrane
U12−type spliceosomal complex
cytosol
U2 snRNP
extracellular exosome
ficolin−1−rich granule lumen
membrane
cytosolic large ribosomal subunit
extracellular exosome
cytosol
ribonucleoprotein complex
polysomal ribosome
cytosolic small ribosomal subunit
focal adhesion
ribosome
cytoplasmic stress granule
extracellular space
nucleosome
SMN complex
extracellular region
basement membrane
Gemini of coiled bodies
cytosolic large ribosomal subunit
extracellular matrix
endoplasmic reticulum lumen
collagen−containing extracellular matrix
c
GO Molecular Function
protease binding
proton−transporting ATPase activity, rotational mechanism
MHC class I protein binding
actin filament binding
enzyme binding
ribose phosphate diphosphokinase activity
protein binding
guanyl−nucleotide exchange factor activity
histone acetyltransferase binding
protein domain specific binding
RNA binding
structural constituent of ribosome
cadherin binding
mRNA binding
mRNA 3'−UTR binding
protein binding
translation initiation factor activity
translation initiation factor binding
mRNA 5'−UTR binding
double−stranded RNA binding
extracellular matrix structural constituent conferring tensile strength
nucleosomal DNA binding
chromatin DNA binding
platelet−derived growth factor binding
extracellular matrix structural constituent
carbohydrate binding
protein heterodimerization activity
extracellular matrix binding
endopeptidase inhibitor activity
protein−lysine 6−oxidase activity
d
GO Biological Process
antigen processing and presentation of exogenous peptide antigen via MHC class I, TAP−dependent
regulation of hematopoietic stem cell differentiation
ciliary basal body−plasma membrane docking
regulation of cellular amino acid metabolic process
proton transmembrane transport
Wnt signaling pathway, planar cell polarity pathway
negative regulation of G2/M transition of mitotic cell cycle
regulation of mitotic cell cycle phase transition
transmembrane transport
T cell receptor signaling pathway
translational initiation
SRP−dependent cotranslational protein targeting to membrane
nuclear−transcribed mRNA catabolic process, nonsense−mediated decay
viral transcription
translation
mRNA splicing, via spliceosome
cytoplasmic translation
formation of cytoplasmic translation initiation complex
negative regulation of translation
regulation of alternative mRNA splicing, via spliceosome
nucleosome assembly
leukocyte migration
extracellular matrix organization
negative regulation of chromatin silencing
chromosome condensation
SRP−dependent cotranslational protein targeting to membrane
translational initiation
collagen fibril organization
Fc−gamma receptor signaling pathway involved in phagocytosis
histone H3−K4 trimethylation

Supplementary Figure 16. Gene set enrichment of the PPI networks calculated using MSigDB Reactome (a), GO CC (b), GO MF (c), and GO BP (d) terms. Interactors found exclusively in the EC (EC only) or SMC (SMC only) network and interactors found in both networks (Intersect) were analyzed separately and compared against the non-interactors detected by IP-MS; only the top 10 gene sets are shown for each analysis. All P-values were calculated using one-tailed hypergeometric tests. Nominally ($P < 0.05$) or Bonferroni-significant ($P <$ 0.05/number of gene sets) results are shown in orange or red, respectively. Gene counts used for analysis are shown in **Supplementary Data 11-12**.

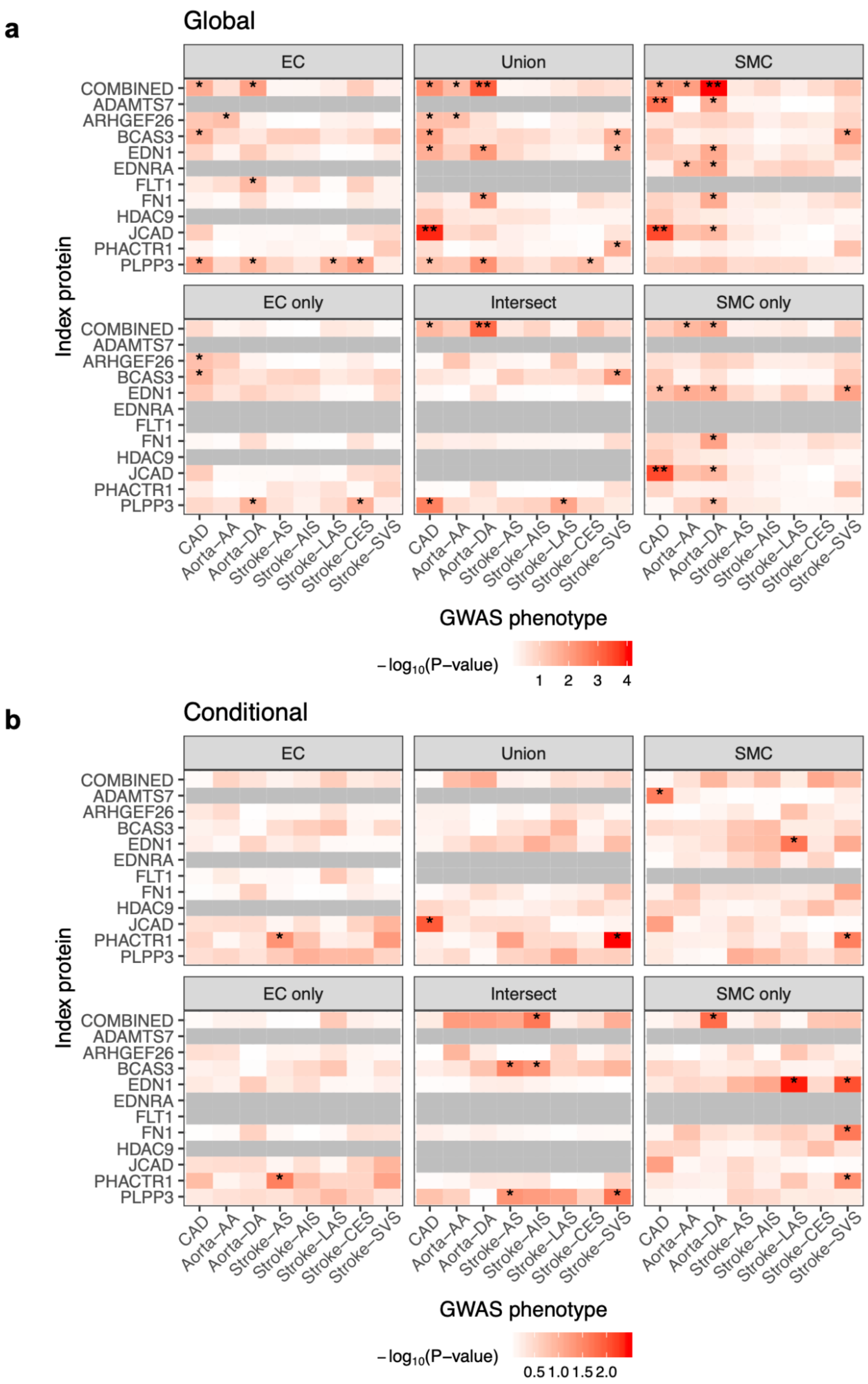

a
Global
EC
Union
SMC
EC only
Intersect
SMC only
COMBINED
ADAMTS7
ARHGEF26
BCAS3
EDN1
EDNRA
FLT1
FN1
HDAC9
JCAD
PHACTR1
PLPP3
Index protein
CAD
Aorta-AA
Aorta-DA
Stroke-AS
Stroke-AIS
Stroke-LAS
Stroke-CES
Stroke-SVS
GWAS phenotype
$-\log_{10}$(P-value)
1 2 3 4
b
Conditional
0.5 1.0 1.5 2.0

Supplementary Figure 17. Common variant enrichment of PPI networks calculated using MAGMA and GWAS summary statistics of CAD, aorta size, and stroke. Enrichment of index protein interactors identified in EC, SMC, either cell type (Union), both cell types (Intersect), exclusively in EC (EC only), or exclusively in SMC (SMC only) were compared against the rest of the protein-coding genome (**a**; Global) or the non-interactors detected in IP-MS experiments (**b**; Conditional). Nominal ($P < 0.05$) or Bonferroni ($P < 0.05/29$, adjusting for 29 EC, SMC, and Union networks) significance is indicated by single or double asterisks, respectively. Gene counts used for analysis are shown in **Supplementary Data 13**. Gray cells in heat maps indicate missing data. AA, ascending aorta; DA, descending aorta; AS, any stroke; AIS, any ischemic stroke; LAS, large-artery atherosclerotic stroke; CES, cardioembolic stroke; SVS, small-vessel stroke.

a

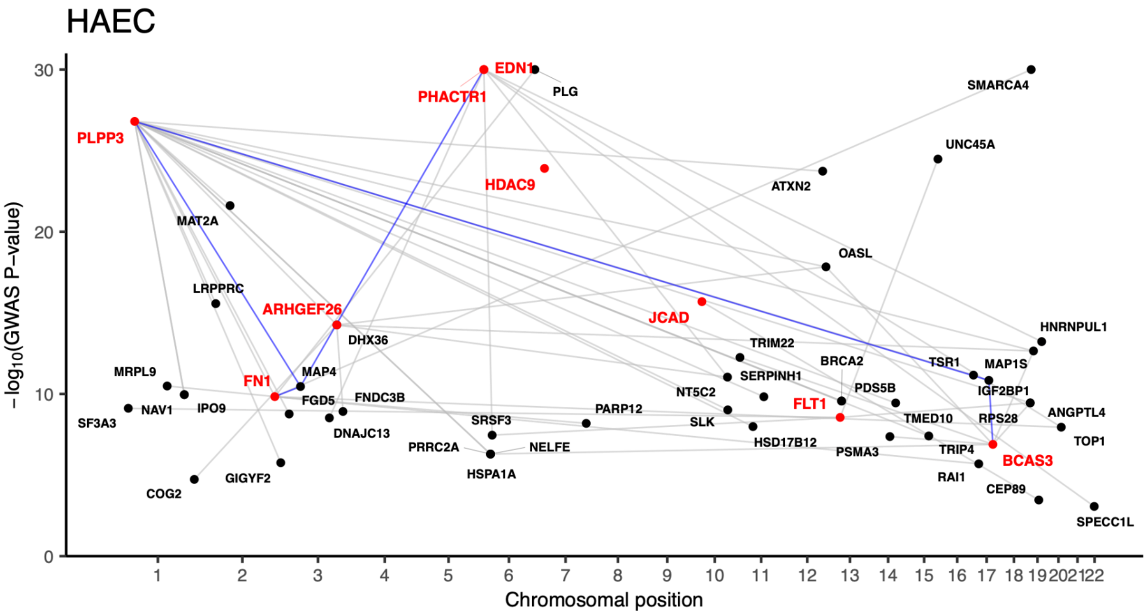

b

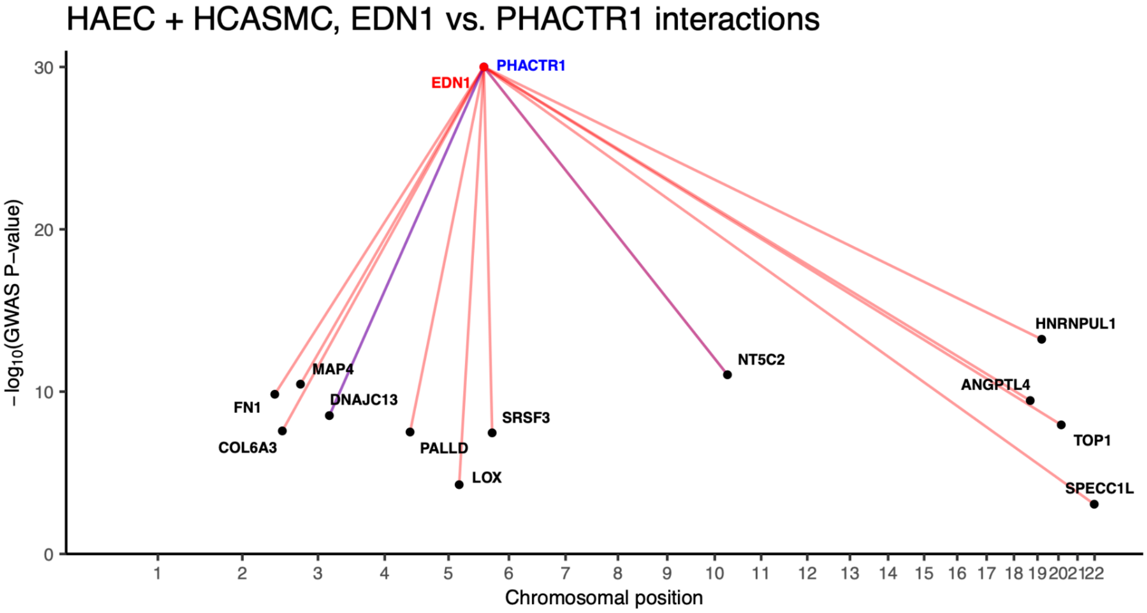

Supplementary Figure 18. Overlap between the PPI networks and genes in CAD GWAS loci. (a) Social Manhattan plot of genes encoding the index proteins (red) and their EC interactors (black) in genome-wide significant CAD GWAS loci. Links between genes indicate observed protein-protein interactions; interactions validated by IP-WB are highlighted in blue. **(b)** Social Manhattan plot of genes encoding EDN1, PHACTR1, and their EC or SMC interactors (black) in genome-wide significant CAD GWAS loci. Links between genes indicate observed protein-protein interactions; EDN1 and PHACTR1 interactions are shown in red and blue, respectively. Both PHACTR1 interactors (DNAJC13 and NT5C2) are also EDN1 interactors.

a Endothelial cell

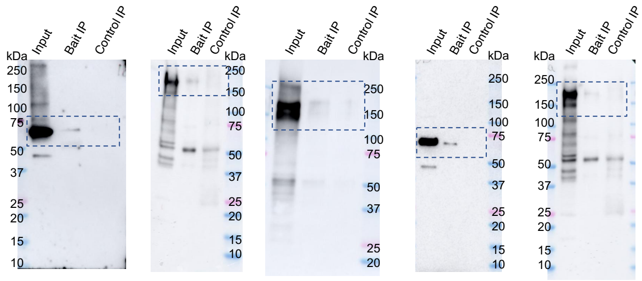

IP	BCAS3	EDN1	FN1	PLPP3	PLPP3
Detection	IGF2BP1 (63 kDa)	MAP4 (210-240 kDa)	MAP4 (210-240 kDa)	IGF2BP1 (63 kDa)	MAP4 (210-240 kDa)

b Smooth muscle cell

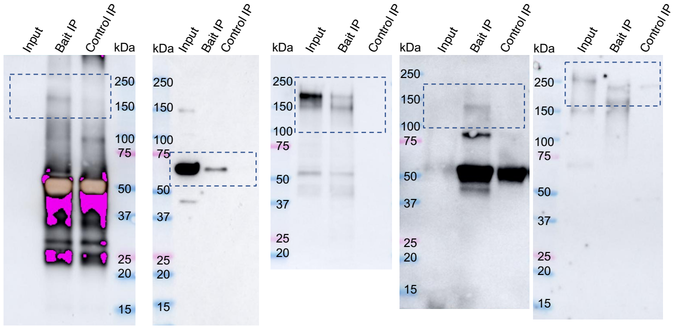

IP	ADAMTS7	ADAMTS7	EDN1	EDNRA	EDNRA
Detection	ATXN2 (140 kDa)	IGF2BP1 (63 kDa)	MAP4 (210-240 kDa)	FNDC3B (133 kDa)	TNS1 (186 kDa)

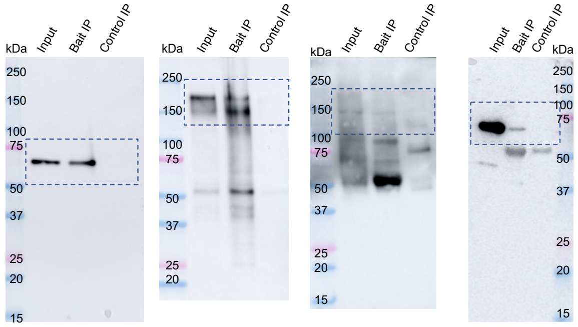

IP	FN1	FN1	JCAD	JCAD
Detection	IGF2BP1 (63 kDa)	MAP4 (210-240 kDa)	FNDC3B (133 kDa)	IGF2BP1 (63 kDa)

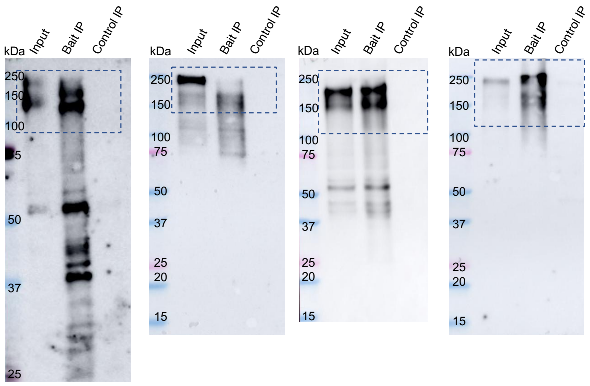

IP	JCAD	JCAD	PLPP3	PLPP3
Detection	MAP4 (210-240 kDa)	TNS1 (186 kDa)	MAP4 (210-240 kDa)	TNS1 (186 kDa)

Supplementary Figure 19. Validation of selected interactors found in CAD GWAS loci. IPs of the index proteins were performed in EC **(a)** or SMC **(b)**, then subjected to Western blot analysis to detect the presence of selected interactors. Control IPs for FLAG-tagged baits were performed using an anti-FLAG antibody in empty-vector-transfected cells; control IPs for endogenous baits were performed using a non-specific rabbit or mouse IgG antibody. Antibodies used in IP and Western blot are listed in **Supplementary Data 6** and **Methods**. Each blot represents one of two IP replicates. Bands corresponding to the expected molecular weight of the Detection protein (shown in parentheses) are marked by dashed boxes.
