# Supplementary material for: Protein interaction networks in the vasculature prioritize genes and pathways underlying coronary artery disease
Source: Commun Biol. 2024 Jan 12;7:87. doi: 10.1038/s42003-023-05705-1 (PMC10786878; doi:10.1038/s42003-023-05705-1)
Supplement: Supplementary file 2 — Description of Additional Supplementary Files [file 42003_2023_5705_MOESM2_ESM.pdf]

Description of Additional Supplementary File

File name: Supplementary Data 1
Description: Source data for main and supplementary figures.

File name: Supplementary Data 2
Description: Pooled CAD-risk loci from genetic association studies.

File name: Supplementary Data 3
Description: Index gene expression in HAEC and HCASMC.

File name: Supplementary Data 4
Description: Genetic and experimental evidence implicating the index genes in CAD.

File name: Supplementary Data 5
Description: Analysis results for the 20 IP-MS datasets that passed QC.

File name: Supplementary Data 6
Description: Summary of the 20 IP-MS datasets that passed QC.

File name: Supplementary Data 7
Description: GO term enrichment analysis of JCAD interactors in EC**.**

File name: Supplementary Data 8
Description: Summary of interactors and non-interactors in the PPI networks.

File name: Supplementary Data 9
Description: Overlap of interactors between each pair of IP-MS datasets.

File name: Supplementary Data 10
Description: Tissue enrichment of the PPI networks calculated using GTEx tissue-specific genes.

File name: Supplementary Data 11
Description: Enrichment of MSigDB Hallmark and Reactome gene sets in the PPI networks.

File name: Supplementary Data 12
Description: Enrichment of GO MF, CC, and BP terms in the PPI networks.

File name: Supplementary Data 13
Description: Common variant enrichment of the PPI networks calculated using MAGMA and GWAS summary statistics.

File name: Supplementary Data 14
Description: Protein interactions between index genes and genes in CAD GWAS loci.

File name: Supplementary Data 15
Description: Overlap between CAD PPI data from this study and CRISPR screen data from Wunnemann et al.
