# Supplementary material for: Protein interaction networks in the vasculature prioritize genes and pathways underlying coronary artery disease
Source: Commun Biol. 2024 Jan 12;7:87. doi: 10.1038/s42003-023-05705-1 (PMC10786878; doi:10.1038/s42003-023-05705-1)
Supplement: Supplementary file 5 — Reporting Summary [file 42003_2023_5705_MOESM5_ESM.pdf]

nature portfolio

Corresponding author(s): Patrick T. Ellinor and Kasper Lage

Last updated by author(s): Oct 4, 2023

Reporting Summary

Nature Portfolio wishes to improve the reproducibility of the work that we publish. This form provides structure for consistency and transparency in reporting. For further information on Nature Portfolio policies, see our Editorial Policies and the Editorial Policy Checklist.

Statistics

For all statistical analyses, confirm that the following items are present in the figure legend, table legend, main text, or Methods section.

n/a	Confirmed	
☐	☒	The exact sample size (*n*) for each experimental group/condition, given as a discrete number and unit of measurement
☐	☒	A statement on whether measurements were taken from distinct samples or whether the same sample was measured repeatedly
☐	☒	The statistical test(s) used AND whether they are one- or two-sided<br>*Only common tests should be described solely by name; describe more complex techniques in the Methods section.*
☒	☐	A description of all covariates tested
☐	☒	A description of any assumptions or corrections, such as tests of normality and adjustment for multiple comparisons
☐	☒	A full description of the statistical parameters including central tendency (e.g. means) or other basic estimates (e.g. regression coefficient) AND variation (e.g. standard deviation) or associated estimates of uncertainty (e.g. confidence intervals)
☐	☒	For null hypothesis testing, the test statistic (e.g. *F*, *t*, *r*) with confidence intervals, effect sizes, degrees of freedom and *P* value noted<br>*Give P values as exact values whenever suitable.*
☒	☐	For Bayesian analysis, information on the choice of priors and Markov chain Monte Carlo settings
☒	☐	For hierarchical and complex designs, identification of the appropriate level for tests and full reporting of outcomes
☐	☒	Estimates of effect sizes (e.g. Cohen's *d*, Pearson's *r*), indicating how they were calculated

Our web collection on statistics for biologists contains articles on many of the points above.

Software and code

Policy information about availability of computer code

Data collection	Mass spectra generated in IP-MS experiments were analyzed by the mass spectrometry facilities using Spectrum Mill (v7.0; https://proteomics.broadinstitute.org) or PEAKS Studio X+ (Bioinformatics Solutions).
Data analysis	IP-MS and tissue/gene set enrichment analyses were performed using the Genoppi R package (v1.0.0). Network plots were generated using the igraph (v1.2.5) and qgraph (v1.6.5) R packages. Genetic risk enrichment analysis was performed using MAGMA (v1.09). LD boundaries for GWAS loci were defined using PLINK (v1.9). Original code has been deposited at GitHub (https://github.com/lagelab/CAD_PPI) and Zenodo (https://doi.org/10.5281/zenodo.8415025).

For manuscripts utilizing custom algorithms or software that are central to the research but not yet described in published literature, software must be made available to editors and reviewers. We strongly encourage code deposition in a community repository (e.g. GitHub). See the Nature Portfolio guidelines for submitting code & software for further information.

Data

Policy information about availability of data

All manuscripts must include a data availability statement. This statement should provide the following information, where applicable:

- Accession codes, unique identifiers, or web links for publicly available datasets
- A description of any restrictions on data availability
- For clinical datasets or third party data, please ensure that the statement adheres to our policy

The mass spectra from IP-MS experiments and the protein sequence databases used for searches have been deposited at MassIVE (https://massive.ucsd.edu) with identifiers MSV000091373 (data from Whitehead Proteomics Core Facility) and MSV000091699 (data from Broad Proteomics Platform). Source data for figures are documented in Supplementary Data 1.

Research involving human participants, their data, or biological material

Policy information about studies with human participants or human data. See also policy information about sex, gender (identity/presentation), and sexual orientation and race, ethnicity and racism.

Reporting on sex and gender	This study does not involve human research participants.
Reporting on race, ethnicity, or other socially relevant groupings	This study does not involve human research participants.
Population characteristics	This study does not involve human research participants.
Recruitment	This study does not involve human research participants.
Ethics oversight	This study does not involve human research participants.

Note that full information on the approval of the study protocol must also be provided in the manuscript.

Field-specific reporting

Please select the one below that is the best fit for your research. If you are not sure, read the appropriate sections before making your selection.

☒ Life sciences ☐ Behavioural & social sciences ☐ Ecological, evolutionary & environmental sciences

For a reference copy of the document with all sections, see nature.com/documents/nr-reporting-summary-flat.pdf

Life sciences study design

All studies must disclose on these points even when the disclosure is negative.

Sample size	No statistical method was used to determine sample size. Instead, replication is more relevant for the in vitro discovery of biochemical interactions reported in this study (see below).
Data exclusions	No data were excluded from the analysis.
Replication	Immunoprecipitation (IP) experiments were performed in triplicate: one replicate was used to run quality control Western blot (WB) analysis, while the other two replicates were submitted for mass spectrometry (MS) analysis. When performing quality control of the IP-MS datasets, we required the Pearson's correlation of log2 fold change values between the two replicates to be > 0.6 for a dataset to be considered in downstream analyses. For a subset of the interactions identified by IP-MS, additional replication/validation was performed through IP-WB analyis as described in the relevant supplementary figures.
Randomization	Each IP-MS experiment/dataset consists of two bait IP samples and two control samples. The samples were labeled with iTRAQ or TMT labels and then pooled together prior to mass spectrometry analysis, thus randomization is not applicable to this experimental design.
Blinding	Sample blinding was not implemented in this study. Statistical analyses were performed to identify the reported protein interactions from the IP-MS/MS data in an unbiased, systematic manner.

Reporting for specific materials, systems and methods

We require information from authors about some types of materials, experimental systems and methods used in many studies. Here, indicate whether each material, system or method listed is relevant to your study. If you are not sure if a list item applies to your research, read the appropriate section before selecting a response.

Materials & experimental systems

n/a	Involved in the study	
☐	☒	Antibodies
☐	☒	Eukaryotic cell lines
☒	☐	Palaeontology and archaeology
☒	☐	Animals and other organisms
☒	☐	Clinical data
☒	☐	Dual use research of concern
☒	☐	Plants

Methods

n/a	Involved in the study	
☒	☐	ChIP-seq
☒	☐	Flow cytometry
☒	☐	MRI-based neuroimaging

Antibodies

Antibodies used

The sources of antibodies and normal IgG for Co-IP are listed in Supplementary Data 6 and below:
Anti-FLAG M2 magnetic beads (Sigma-Aldrich, M8823)
Anti-FLAG M2 affinity gel (Sigma-Aldrich, A2220)
Normal mouse IgG (Santa Cruz sc-2343)
Normal rabbit IgG (R&D Systems, AB-105-C)
Anti-FLAG M2-Peroxidase (HRP) antibody (Sigma-Aldrich, A8592)
Rabbit IgG HRP-conjugated antibody (R&D Systems, HAF-008)
Mouse IgG HRP-conjugated antibody (R&D Systems, HAF-007)
Anti-ADAMTS7 (Abcam, Ab28557)
Anti-ATXN2 (Novus Biologicals, NBP1-90063)
Anti-EDNRA (Abcam, ab242440)
Anti-FLT1 (Santa Cruz, sc-271789)
Anti-FLT1 (Thermo Fisher Scientific, PA5-16493)
Anti-FN1 (Santa Cruz, sc-8422 AC)
Anti-FN1 (Sigma-Aldrich, AB1945)
Anti-FNDC3B (Novus Biologicals, NBP1-90495)
Anti-HSPA9 (Cell Signaling Technology, 3593T)
Anti-IGF2BP1 (Cell Signaling Technology, 8482)
Anti-JCAD (Sigma-Aldrich, HPA017956)
Anti-MAP4 (Proteintech, 11229-1-AP)
Anti-PDIA6 (Sigma-Aldrich, HPA034652)
Anti-RPL7A (Cell Signaling Technology, 2415)
Anti-TNS1 (Novus Biologicals, NBP1-84130)

Validation

All antibodies used in this study have been validated by the respective vendors as cited above. In addition to validation statements on the manufacturers websites and data sheets, we have performed validation Co-IP and western blot experiments to confirm the specificity and competency for Co-IP. The validation results are shown in Supplemental Figures 1 and 2.

Eukaryotic cell lines

Policy information about cell lines and Sex and Gender in Research

Cell line source(s)

HAEC (Cat #FC-0014) and HCASMC (Cat #FC-0031) from multiple healthy donors were pooled from the manufacturer (Lifeline Cell Technology), including both male and female donors. HEK293 cell was from ATCC (Cat #CRL-1573), gender unknown, reported as having three copies of X chromosomes.

Authentication

Extensive authentications have been performed by the manufacturers above and described on manufacturers websites.

Mycoplasma contamination

Cell lines were tested negative for mycoplasma contamination as per cell culture protocols of the Broad Institute.

Commonly misidentified lines
(See ICLAC register)

No commonly misidentified cell lines were used.
